# Reproducibility and Validity of a Nova-Based Food Frequency Questionnaire in Older Italian Adults: The NFFQ-Elderly

**DOI:** 10.3390/nu18081266

**Published:** 2026-04-16

**Authors:** Annarita Formisano, Marika Dello Russo, Emilia Ruggiero, Giuseppe Di Costanzo, Marialaura Bonaccio, Licia Iacoviello, Pasquale Marena, Fabio Lauria

**Affiliations:** 1 Institute of Food Science, National Research Council of Italy, 83100 Avellino, Italy; marika.dellorusso@isa.cnr.it (M.D.R.); pasquale.marena@isa.cnr.it (P.M.); 2 Research Unit of Epidemiology and Prevention, IRCCS NEUROMED, 86077 Pozzilli, Italy; emilia.ruggiero@neuromed.it (E.R.); giuseppe.dicostanzo@neuromed.it (G.D.C.); marialaura.bonaccio@neuromed.it (M.B.); licia.iacoviello@neuromed.it (L.I.); 3 Department of Medicine and Surgery, LUM University, 70010 Casamassima, Italy

**Keywords:** Nova classification, food frequency questionnaire, ultra-processed foods, older adults, validation, reproducibility, dietary assessment

## Abstract

**Background/Objectives:** Nutritional research emphasizes evaluating food processing levels alongside nutrient content. The Nova system categorizes foods as minimally processed foods (MPFs), processed culinary ingredients (PCIs), processed foods (PFs), and ultra-processed foods (UPFs). High UPF consumption is linked to adverse health outcomes in older adults. Traditional Food Frequency Questionnaires (FFQs) often fail to capture processing differences. This study evaluated the reproducibility and relative validity of a Nova-based FFQ (NFFQ-Elderly) in Italian healthy older adults aged ≥65 years. **Methods:** A total of 111 older adults (73.7 ± 5.9 years; 56.8% women) completed the NFFQ-Elderly twice (4–6 weeks interval). Relative validity was compared with a three-day weighed food record. Foods were categorized by Nova groups and analyzed for absolute intake, energy and weight percentages. Pearson correlation (*r*), intraclass correlation coefficients (ICCs), and Bland–Altman plots were used. **Results:** Reproducibility was satisfactory for MPFs (*r* = 0.75; ICC = 0.74), UPFs (*r* = 0.87; ICC = 0.85), and PFs (*r* ≈ 0.73; ICC ≈ 0.66–0.67). Relative validity was moderate for MPFs (*r* = 0.57; ICC = 0.53) and UPFs (*r* = 0.48; ICC ≈ 0.37), but lower for PCIs. Accuracy generally improved when intakes were expressed as percentages of total energy or weight. Bland–Altman analyses showed limited mean bias for MPFs and PFs, but higher variability for PCIs and absolute energy intake. **Conclusions:** The NFFQ-Elderly appears to be a suitable tool for ranking older adults according to their relative intake of MPFs and UPFs. Estimates for PCIs are less reliable, indicating caution when interpreting absolute intake values.

## 1. Introduction

In recent years, a growing body of evidence has highlighted the need to move beyond traditional approaches focused solely on nutrient composition, advocating instead for an integrated assessment of the degree and nature of industrial food processing [1,2,3]. This shift reflects the recognition that food processing can alter the food’s structure, sensory characteristics, energy density, and consumption patterns. Furthermore, it accounts for the presence of additives or contaminants, all factors that may influence satiety, nutrient bioavailability, and the risk of chronic diseases [4,5]. Among the various classification systems proposed internationally [6,7], the Nova classification, introduced by Monteiro et al. [8], has become the most widely used framework in epidemiological research. Nova categorizes foods according to the extent and purpose of industrial processing, incorporating four key dimensions: degree, nature, place, and purpose of processing [9]. By providing a detailed understanding of how industrial transformations affect food characteristics and health outcomes [9], the system has established itself as a benchmark in nutritional research. Specifically, it categorizes foods into four distinct groups: unprocessed or minimally processed foods (MPFs), processed culinary ingredients (PCIs), processed foods (PFs), and ultra-processed foods (UPFs) [10,11]. This classification facilitates the characterization of overall dietary patterns within contemporary food systems and consumption behaviors.

Several studies have consistently reported strong associations between high UPF consumption and adverse health outcomes. Evidence from cross-sectional and prospective cohort studies links UPF intake to higher risk of non-communicable diseases (NCDs), including obesity, type 2 diabetes, dyslipidemia, and hypertension [12,13,14,15]. Moreover, rising UPF consumption has been associated with a progressive reduction in overall diet quality across several countries [16,17]. This issue is of particular concern in older adults, among whom higher UPF intake has been associated with cognitive decline, poor executive function [18], increased dementia risk [19], and worsening kidney function [20]. In contrast, diets rich in unprocessed or minimally processed foods are linked to more favorable health profiles.

Despite the widespread adoption of the Nova classification in nutritional research, methodological challenges remain in the accurate assessment of food consumption according to processing levels. Commonly used dietary assessment tools were not designed to distinguish between homemade, artisanal, and industrially processed foods. This limitation increases the risk of misclassification and highlights the need for instruments specifically developed to capture processing-related details [21,22,23,24].

To date, only a few Food Frequency Questionnaires (FFQs) have been specifically developed and validated for this purpose. In Italy, a Nova-based tool has been validated in the adult population by Dinu et al. [25], while more recently Paladino et al. validated a similar questionnaire for children and adolescents in Southern Italy [26]. However, to our knowledge, no tools have been specifically adapted for older adults. This research gap is significant, as this demographic presents specific dietary characteristics that may not be adequately captured by instruments designed for younger adults [18,27]. Age-related changes in appetite, chewing ability, and meal patterns affect both the types of foods consumed and typical portion sizes. Furthermore, older individuals in Italy often maintain traditional eating patterns with a higher proportion of home-prepared dishes, which require detailed descriptions to be correctly classified under the Nova framework [28,29]. Additionally, differences in cognitive burden and recall accuracy further necessitate simplified wording and concrete examples to improve comprehension and reduce misclassification [30].

Building on the Nova FFQ by Dinu et al. [25], the present study aims to adapt and validate this tool for healthy older Italian adults (≥65 years).

## 2. Materials and Methods

### 2.1. Study Design, Participants, and Ethical Considerations

The validation study was conducted within the framework of the NUTRAGE project (Nutrition, Diet & Active Aging), funded by the Italian National Research Council (CNR) through FOE 2021 funds, aimed at promoting healthy lifestyles in the population aged ≥65 years, addressing emerging issues related to aging and chronic diseases.

Recruitment took place between February and June 2025 in the Campania region (Southern Italy). A total of 117 individuals were randomly selected through local associations focused on cultural, educational, and social activities for older adults. Of these, 111 participants (age 73.7 ± 5.9 years; 56.8% women) provided valid dietary data and were included in the final analysis. The sample size is consistent with Willett’s recommendation that validation studies typically require 100–200 participants to obtain correlation estimates with adequate precision [30].

Individuals with medical conditions requiring specific diets (e.g., diabetes, renal failure, gastrointestinal disorders), those with conditions affecting dietary intake or cognitive function, and those who had followed specific diets or weight-loss programs in the previous 12 months were excluded.

The study was conducted in accordance with the ethical principles of the Declaration of Helsinki and complied with relevant national and European regulations on research ethics and data protection (including Regulation EU 2016/679-GDPR). Ethical approval was granted by the Ethics and Research Integrity Committee of the CNR of Italy (Protocol No. 0002560/2024, 18 November 2024). All participants provided written informed consent prior to enrolment. This study is reported in accordance with the Strengthening the Reporting of Observational Studies in Nutritional Epidemiology (STROBE-nut) checklist, an extension of the STROBE statement (Supplementary Table S1) [31].

### 2.2. Data Collection Procedures

Participants were recruited through informational meetings, open to the public, in the municipalities of Avellino, Meta, Torre del Greco and Vico Equense (Campania region, Southern Italy), during which the study objectives, procedures, data protection measures, and voluntary nature of participation were explained. A preliminary pilot phase was conducted with a representative group of ten older adults (≥65 years) to assess the clarity, usability, and overall comprehension of the Nova-based FFQ specific for older adults (NFFQ-Elderly), enabling further refinement of the tool.

**Figure 1:**
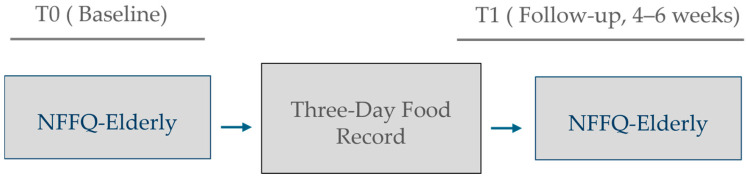
Timeline of questionnaire administration. NFFQ-Elderly at baseline (T0) and follow-up (T1); three-day FR completed at least one week after baseline as the reference method.

Participants completed NFFQ-Elderly, either online via a personalized link sent by e-mail or in paper format, according to their digital literacy and personal preference. For those who chose the paper format, the completed questionnaires were manually entered by trained researchers into a secure electronic database, and each participant was assigned a unique alphanumeric ID code to ensure anonymity and traceability. The NFFQ-Elderly was administered at baseline and again after 4–6 weeks to evaluate its reproducibility, as shown in Figure 1. To assess the relative validity of the NFFQ-Elderly, a three-day Food Record (FR) was employed as the reference method and completed by participants. Recording days were randomly selected to better reflect habitual intake and reduce systematic bias, balancing data quality with participant burden, particularly considering the older age of the sample.

### 2.3. Dietary Assessment Methods

#### 2.3.1. NFFQ-Elderly

NFFQ-Elderly is a semi-quantitative FFQ designed to estimate habitual dietary intake in Italian adults aged ≥65 years, according to the Nova classification system. It was adapted from the original Nova FFQ validated by Dinu et al. for adults, retaining the overall structure while introducing age-specific modifications to better capture habitual food consumption and nutritional patterns in older adults [25]. The Italian version of the adapted NFFQ-Elderly (a) and the English translation (b) can be found in Supplementary Table S2.

The questionnaire includes 94 items organized into nine food groups: (1) fruit and nuts, (2) vegetables and legumes, (3) cereals and tubers, (4) meat and fish, (5) milk, dairy products, and eggs, (6) oils, fats, and seasonings, (7) sweets and sweeteners, (8) beverages, and (9) other foods. The questionnaire also includes additional frequently consumed foods not listed in the nine main food groups. Participants report the frequency of consumption of foods and beverages over the previous 12 months using ten predefined categories, ranging from “never or less than once a month” to “every day (how many times per day?)”, and indicate usual portion size using six options from 0.5 to 3 standard servings. Portion sizes are expressed in grams, milliliters, or household units, derived from the official Italian recommendations [28,29], or when unavailable, from product packaging information.

The item list was revised to reflect foods commonly consumed by older adults, introducing new items and reorganizing existing ones to improve the capture of nutritional information. In the “Fruit and Nuts” and “Vegetables and Legumes” sections, fruit and vegetable preparations in homogenized form (both homemade and industrial) were included, and fresh and dried legumes were combined into a single item. In the “Cereals and tubers” section, fresh and dried pasta were merged into a single item. Lasagna was grouped with stuffed pasta, while couscous and semolino were combined with fresh and dried pasta. Gnocchi were removed as a separate item, and potatoes and croquettes were split into artisanal/homemade and industrial products.

The “Meat and Fish” section was expanded to include packaged ready-to-eat dishes (e.g., mussels in tomato sauce, marinated salmon filets, seafood salad, spicy grilled chicken), with specific examples to aid recognition.

In the “Milk, Dairy Products, and Eggs” section, ricotta (sheep and cow) was added as a distinct item alongside the existing classification of hard, soft, and fused cheeses, improving the estimation of dietary patterns in older adults.

In the ‘Sweets and Sweeteners’ section, sugar was separated from artificial sweeteners.

Some food item labels were revised to enhance clarity and align with terminology more familiar to older adults. For example, the item originally labeled “meal replacement shakes and powders” was updated to “beverages, smoothies, and other meal replacement products (including bars), including powdered forms.” Special attention was given to distinguishing homemade, artisanal, and industrial preparations, which is crucial for accurate Nova classification, particularly for UPFs. The wording and layout were also simplified and supplemented with concrete examples to facilitate comprehension and accurate reporting.

Food items and ingredients were classified according to the Nova framework [1], following the methodological approach of Dinu et al. [25] and Paladino et al. [26]. MPFs included natural foods or those undergoing minimal processing, such as washing, milling, chilling, freezing, or vacuum-packing. PCIs included items primarily used in cooking, such as oils, fats, sugar, and salt. PFs, typically obtained by combining MPFs and PCIs, were analyzed both as an independent Nova category and in combination with PCIs (PCIs + PFs), in order to account for their joint contribution to home-prepared dishes and to ensure comparability with previous validation studies. UPFs included industrial formulations with multiple ingredients (typically five or more), often containing additives, preservatives, or flavor enhancers, designed to be highly palatable, ready-to-eat, and convenient.

Finally, a section was added to collect sociodemographic and anthropometric data (age, sex, education level, household size, family income, body weight, and height), which was not directly included in the original adult version of the questionnaire. Educational level was classified into four categories: primary, lower secondary, upper secondary, and postsecondary, corresponding to the ISCED 2011 levels relevant for our sample [32]. Household income was categorized into three groups: ≤20,000 EUR/year, 20,000–30,000 EUR/year, and ≥30,000 EUR/year, with non-respondents or missing data recorded separately.

#### 2.3.2. Three-Day Food Record (FR)

To assess the relative validity of the NFFQ-Elderly, a three-day FR was employed as the reference method and completed by participants. Each subject was asked to meticulously document all foods and beverages consumed over three non-consecutive days, including two weekdays and one weekend day, during the week following the first questionnaire administration. The recording days were divided into six eating occasions: breakfast, mid-morning snack, lunch, afternoon snack, dinner, and other.

Participants recorded the name of each food item (including brand when possible), and the amount consumed, expressed in grams (g), milliliters (mL), or household measures (e.g., teaspoons, tablespoons, or standard portions). For items reported in grams, subjects specified the measured weight and whether the food was raw or cooked, with a preference for raw weight whenever possible. They also indicated type of preparation using a standardized coding system with four categories: F (fresh), I (industrial), A (artisanal), and C (homemade). Special attention was given to the documentation of homemade recipes, with individual ingredients and their quantities recorded, as well as added condiments, extra snacks, and commonly overlooked beverages such as water, tea, or coffee.

Detailed written and verbal instructions were provided, alongside supporting materials to assist portion size estimation through household measuring tools. Completed three-day FR were reviewed by trained dietitians for completeness and accuracy. Data were processed using MetaDieta software (version 4.5; METEDA S.r.l., Rome, Italy), based on the Italian Food Composition Tables [29]. For foods not included in the tables, nutrient values were obtained from product labels or manufacturer information. All recorded foods were further classified according to the Nova system, relying on participant-reported information about ingredients, brand, preparation methods, and packaging. This approach ensured methodological consistency between dietary data collected via the three-day FR and those obtained through the NFFQ-Elderly.

To minimize the influence of extreme values, an outlier-based approach was applied to the reported energy intake. Dietary intake data are known to be positively skewed and to present extreme values in the upper tail of the distribution, which may disproportionately affect statistical analyses [33]. Therefore, values below the 1st percentile or above the 99th percentile of the energy intake distribution were classified as implausible and excluded from the analyses.

### 2.4. Statistical Analysis

The sample size was determined a priori to ensure a statistical power of 80% (1 − *β* = 0.80) with a two-tailed *α* level of 0.05. Based on literature concerning the validation of food groups through semi-quantitative FFQs, a moderate correlation coefficient (*r* = 0.50) was anticipated, requiring a minimum of 82 participants (Fisher’s z transformation).

All statistical analyses were conducted using SAS/STAT software, version 9.4 (SAS Institute Inc., Cary, NC, USA), with *p* < 0.05 considered statistically significant. Continuous variables are reported as mean ± standard deviations (SD), and categorical variables as percentages.

The NFFQ-Elderly’s performance in classifying foods according to the Nova system was evaluated in terms of both temporal stability and relative validity. Reproducibility over time (test–retest reliability) was assessed by comparing responses collected at baseline (T0) and 4–6 weeks later (T1), a time interval chosen to reduce recall bias while minimizing potential changes in habitual dietary patterns. Pearson correlation coefficients (*r*) and intraclass correlation coefficients (ICCs) with 95% confidence intervals were calculated via a two-way mixed-effects, absolute agreement model to quantify the stability of reported intakes. Relative validity was determined by comparing baseline NFFQ-Elderly estimates with dietary intake recorded in three-day FR, after excluding participants with implausible energy intakes. Agreement between the NFFQ and the reference method was further examined using Bland–Altman plots for each of the four Nova food categories, with upper and lower limits of agreement to identify potential systematic differences and the magnitude of bias. For each Nova category, intakes were analyzed in grams per day as well as in terms of energy and weight percentages relative to total daily intake. All analyses were performed for the full sample and stratified by sex to explore potential sex-specific patterns. Correlations and agreement metrics were interpreted according to commonly accepted standards, ranging from poor to excellent.

## 3. Results

Of the 117 participants initially recruited, 5.1% (*n* = 6) were excluded from the validation analyses due to incomplete or implausible dietary data. Specifically, two participants did not complete the three-day FR, and four reported implausible energy intakes in either the FR or the NFFQ-Elderly. The final validation sample therefore consisted of 111 participants (73.7 ± 5.9 years; 56.8% women). This sample size provided a post hoc statistical power of 88% to detect the hypothesized correlation of 0.50 (alpha = 0.05, two-tailed).

The mean age was 72.6 ± 5.7 years for women and 75.1 ± 5.9 years for men. Men were slightly older and had a higher BMI compared with women. As shown in Table 1, educational level and household income showed a heterogeneous distribution across the sample, and most participants lived with one other person. Test–retest reliability analyses were conducted among the 110 participants who completed both baseline (T0) and follow-up (T1) administrations of the NFFQ-Elderly.

### 3.1. Preliminary Testing

Preliminary testing of the NFFQ-Elderly indicated high clarity and usability. Most participants (9/10) considered the instructions clear and the questions easy to understand, highlighting the usefulness of examples and simplified wording. Six out of ten completed the questionnaire without rereading any items, and only one participant needed to review some questions. Eight out of ten judged the questionnaire length appropriate, confirming its overall clarity, acceptability, and usability.

### 3.2. Questionnaire Validity

Relative validity analyses were performed in participants with complete NFFQ and three-day FR data, as shown in Table 2. The NFFQ-Elderly demonstrated moderate-to-good validity for the main Nova food groups. Absolute intakes (g/day) estimated by the NFFQ were generally higher than those recorded in the FR, consistent with the typical overestimation observed in FFQs. This pattern was also evident for total energy intake (1902.2 vs. 1577.5 kcal/day). Nevertheless, a significant correlation was maintained for total energy (*r* = 0.40, *p* < 0.0001). Correlation coefficients for absolute intakes ranged from *r* = 0.21 to 0.57, with the highest values observed for MPFs (*r* = 0.57; *p* < 0.0001; ICC = 0.53, 95% CI: 0.39–0.65) and PFs (*r* = 0.41, *p* < 0.0001; ICC = 0.34, 95% CI: 0.20–0.52). UPFs showed moderate correlations (*r* = 0.48; *p* < 0.0001; ICC = 0.21, 95% CI: 0.09–0.44). PCIs were the least accurately reported, with low and marginally significant correlations (*r* = 0.21, *p* = 0.025) and very low reliability (ICC = 0.05, 95% CI: 0.00–0.76).

Relative measures, expressed as energy and weight ratios, showed improved performance for several Nova categories. MPFs displayed higher correlations (*r* = 0.48–0.60, all *p* < 0.0001), with ICC values ranging from 0.48 to 0.53 (95% CI: 0.36–0.62). PFs also showed stronger associations when using relative measures (*r* = 0.37–0.53, *p* < 0.001; ICC = 0.23–0.53, 95% CI: 0.10–0.66). UPFs remained moderately correlated (*r* ≈ 0.45–0.46, *p* < 0.0001; ICC = 0.37–0.47, 95% CI: 0.22–0.62). In contrast, PCIs again showed poor validity, with non-significant correlations for energy ratios (*r* = 0.10, *p* = 0.30).

**Figure 2:**
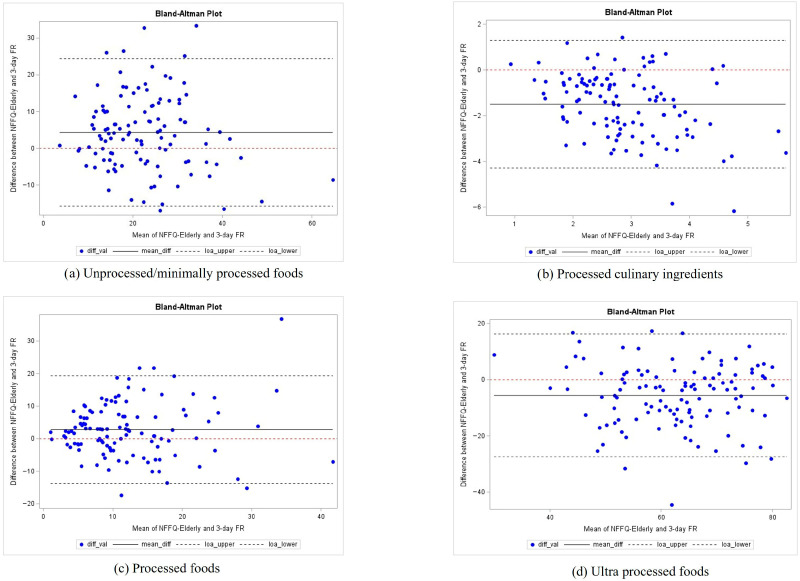
Bland–Altman plots. Comparison of weight ratios for each Nova food group between the NFFQ-Elderly and the three-day FR. The solid line indicates the mean difference, and the dashed lines represent the 95% limits of agreement.

Systematic bias was further assessed through Bland–Altman analyses, which indicated small mean biases across all Nova categories, as illustrated in Figure 2. Specifically, Figure 2a,c demonstrate that MPFs and PFs showed acceptable agreement with moderate variability. While UPFs were slightly underestimated by the NFFQ-Elderly in Figure 2d, the smallest mean differences were displayed by PCIs in Figure 2b.

Sex-stratified analyses, detailed in Supplementary Tables S3 and S4, showed slightly higher correlations and ICCs in men for MPFs and PFs, whereas validity for PCIs remained low in both sexes.

The NFFQ-Elderly appears adequate for classifying older adults according to habitual consumption of Nova food groups, supporting its applicability in epidemiological studies on diet and aging.

### 3.3. Test–Retest Reliability

Temporal stability was evaluated in participants who completed both baseline (T0) and follow-up (T1) administrations of NFFQ-Elderly (Table 3). The questionnaire demonstrated excellent temporal stability across most Nova food groups. For absolute intakes (g/day), correlation coefficients ranged from 0.39 to 0.87 (all *p* < 0.0001) with the highest reliability observed for UPFs (*r* = 0.87 *p* < 0.0001; ICC = 0.85, 95% CI: 0.80–0.90) followed by MPFs (*r* = 0.75, *p* < 0.0001; ICC = 0.74, 95% CI: 0.65–0.81). PFs and the combination of PFs plus PCIs showed good reliability (*r* = 0.73, both *p* < 0.0001; ICC = 0.66–0.67, 95% CI: 0.55–0.76). In contrast, PCIs exhibited the lowest reliability (*r* = 0.39, *p* < 0.0001; ICC = 0.33, 95% CI: 0.19–0.51), consistent with the relative validity findings.

Analyses of relative intake measures (energy and weight ratio) showed similarly strong reliability. MPFs yielded ICC values of 0.80 (95% CI: 0.73–0.86) for energy ratio and 0.82 (95% CI: 0.75–0.87) for weight ratio. UPFs also showed high reliability (ICC = 0.79–0.81, 95% CI: 0.71–0.87; all *p* < 0.0001). PFs demonstrated good reliability across relative measures (ICC = 0.69–0.73, 95% CI: 0.58–0.81), whereas PCIs again showed low consistency, with correlations that were statistically significant but weak (energy ratio: *r* = 0.36, *p* = 0.0001; ICC = 0.29, 95% CI: 0.15–0.48; weight ratio: ICC = 0.21, 95% CI: 0.08–0.44).

Sex-stratified analyses, presented in Supplementary Tables S5 and S6, showed slightly higher reliability in men for unprocessed and PFs, while PCIs remained the least stable category in both sexes.

## 4. Discussion

In the present study, we adapted and validated NFFQ-Elderly, a semi-quantitative food frequency questionnaire designed to estimate habitual dietary intake according to the Nova classification in Italian healthy older adults aged ≥65 years. Our results suggest that the questionnaire is an acceptable and useful tool for epidemiological purposes, showing a moderate capacity to rank participants according to the relative consumption of MPFs, PFs, and UPFs. These findings support its potential application in nutritional research.

Overall, NFFQ-Elderly demonstrated moderate relative validity, with higher correlations for MPFs (*r* = 0.57; ICC = 0.53) and PFs (*r* = 0.41) and lower values for PCIs (*r* = 0.21; ICC ≈ 0.05). The UPFs category showed moderate correlation (*r* = 0.48; ICC ≈ 0.37), which is consistent with previous validation studies of Nova-based FFQs in Italian adults [25] and international populations [34]. As observed in other validations efforts, FFQs tend to overestimate absolute intake compared with weighed FRs or recalls likely due to memory errors, extensive food lists, and difficulties in portion size estimation [25,30]. In our study, the mean daily intakes estimated by the NFFQ-Elderly were generally higher than those recorded in the three-day FR, reflecting a systematic overestimation of total energy intake (1902.2 vs. 1577.5 kcal/day). Bland–Altman analyses confirmed limited mean bias for MPFs and PFs, moderate variability for UPFs, and greater variability for PCIs and total energy. Consequently, we acknowledge that this systematic bias invalidates the use of the instrument for assessing absolute caloric or nutritional adequacy at the individual level. However, while absolute quantification should be avoided, NFFQ-Elderly demonstrated an acceptable ability to rank participants according to the relative intake of NOVA groups, which remains the primary objective in dietary epidemiology studies [25,30,34]. Furthermore, a significant correlation was observed for total energy (r = 0.40, *p* < 0.0001), supporting the questionnaire’s suitability for comparative analyses and for investigating associations between dietary patterns and health outcomes.

The choice of reference method may influence validity estimates. Studies using single 24 h recalls as the gold standard tend to produce lower correlations compared to those with multi-day FRs due to limited capture of day-to-day dietary variability. Nevertheless, these estimates are generally adequate for classifying individuals into relative intake categories [35]. In this context, our use of a three-day FR likely reduced misclassification due to day-to-day variability, providing more robust estimates of habitual intake. This methodological difference may partly explain variability in correlation coefficients across studies using different reference methods.

The NFFQ-Elderly also demonstrated satisfactory test–retest reproducibility over a 4–6-week interval, with strong correlations for MPFs (*r* = 0.75; ICC = 0.74) and UPFs (*r* = 0.87; ICC = 0.85) and good reproducibility for PFs (*r* ≈ 0.73; ICC ≈ 0.66–0.67). PCIs again showed lower stability, reflecting the inherent difficulty of accurately reporting oils, sugars, and condiments in homemade preparations. The use of relative measures (percentage of energy or weight) further improved agreement, suggesting the questionnaire’s effectiveness in ranking individuals according to relative consumption rather than estimating absolute intakes, consistent with recommendations in nutritional epidemiology.

These findings are largely in line with previous evidence from Nova-based FFQs in Italian and international adult populations. Dinu et al. [25] reported good reproducibility and acceptable validity of a Nova FFQ in Italian adults, with higher correlations for MPFs and UPFs, and lower values for PCIs, a pattern closely mirroring our results. International validation studies have similarly reported moderate correlations for MPFs and UPFs (Pearson r typically ranging from 0.3 to 0.7), with consistently lower validity for processed culinary ingredients [25,34]. Sex-stratified analyses, provided in Supplementary Tables S3–S6, offer additional insight into the tool’s performance across genders. Although differences were modest, slightly higher reliability and validity coefficients in men for MPFs and PFs may reflect sex-related differences in dietary variability or reporting accuracy, as suggested in the dietary assessment literature. Nevertheless, the NFFQ-Elderly appears adequate to rank participants of both sexes across the main Nova categories, supporting its potential applicability in gender-balanced epidemiological studies. Although conducted in younger populations, the study by Paladino et al. [26] supports a methodological pattern consistently observed across age groups. Specifically, the intrinsic difficulty in accurately capturing PCIs using FFQs suggests that lower validity for this group reflects structural limitations of dietary assessment tools rather than population-specific factors.

From an epidemiological perspective, the development of validated tools to assess UPF intake in older adults is particularly relevant, given the growing evidence linking high UPF consumption to adverse health outcomes [18,19,36]. Prospective cohort studies further support these associations, showing that higher UPF intake was prospectively linked to greater risk of frailty and functional decline in older adults [27,37].

Additionally, population-level surveys indicate that UPFs contribute substantially to total energy intake in older adults. In a representative sample of middle-aged and older US adults, approximately 50% of daily energy came from UPFs, with higher consumption associated with unfavorable nutrient profiles, including increased intake of added sugars and saturated fats, and reduced fiber and micronutrient density [38,39]. These findings underscore the public health relevance of accurately quantifying UPF consumption in aging populations. NFFQ-Elderly therefore addresses a methodological gap, enabling a reliable ranking of habitual UPF intake and supporting epidemiological research on diet health relationships in adults aged ≥65 years.

Key strengths of this study include the use of a three-day FR as a reference method, which captures day-to-day variability in dietary intake; the cultural and linguistic adaptation of the questionnaire for Italian older adults, including concrete examples and age-appropriate portions; and the comprehensive evaluation of questionnaire performance, encompassing relative validity, test–retest reproducibility, and analyses of both absolute and relative intakes. These features provide methodological robustness and suggest that NFFQ-Elderly is a potentially suitable tool for nutritional and public health research in older populations.

Despite these strengths, several limitations must be acknowledged. The Nova classification of certain foods, particularly mixed dishes and complex home-prepared recipes, can be subjective and dependent on the researcher’s interpretation, as highlighted in previous studies on Nova-based assessments; this may introduce non-systematic variability in the estimation of Nova groups.

More significantly, the low validity of PCIs (r = 0.21; ICC = 0.05) reflects the inherent challenge of accurately recalling discretionary condiments, potentially propagating measurement error to composite dishes. However, aggregating PCIs with Processed Foods (PCIs + PFs) yielded a robust compensatory effect, substantially improving all validity parameters. This combined approach effectively mitigates the recall bias associated with distinguishing added culinary ingredients from those intrinsic to processed items. Consequently, while isolated PCI estimates require caution, the PCIs + PFs aggregation provides a reliable metric. Ultimately, NFFQ-Elderly demonstrates an acceptable capacity to rank participants across the main food groups (MPFs, PFs, and UPFs), confirming its suitability for comparative dietary epidemiology. Furthermore, the overestimation of absolute intake, although common in FFQs, indicates that NFFQ-Elderly is better suited for ranking individuals into relative categories rather than providing precise estimates of grams or calories. Regarding external validity, the study sample was recruited exclusively from a specific geographic area (Campania, Italy) and included generally healthy older adults. These factors represent a direct threat to the generalizability of our findings, and caution is needed when applying NFFQ-Elderly to older populations with different health conditions or dietary habits. Additionally, the cross-sectional design of the study does not allow assessment of the FFQ’s sensitivity to temporal changes in dietary intake, which could be relevant in longitudinal studies of diet and health. Finally, a methodological mismatch exists between the 12-month recall period of NFFQ-Elderly and the three-day FR used as a reference. While the three-day FR is a robust tool for short-term intake, it may not fully capture the intra-individual variability and seasonal nuances reflected in a long-term FFQ. This temporal discrepancy is a recognized source of attenuation in correlation coefficients and likely contributes to the imperfect agreement observed between the two instruments.

## 5. Conclusions

In conclusion, NFFQ-Elderly appears to be an acceptable and practical tool for ranking Italian older adults according to their habitual consumption of food groups defined by the Nova classification. The instrument demonstrated satisfactory reproducibility and moderate relative validity for the main food groups (MPFs, PFs, and UPFs), suggesting its potential application in epidemiological research aimed at investigating dietary patterns and health outcomes in older populations. However, the observed systematic overestimation of total energy intake and the limited validity for PCIs indicate that the tool is not intended for precise absolute quantification of dietary intake. Furthermore, its application to populations outside of the specific geographic and healthy profile of the study sample should be approached with caution. Despite these inherent limitations of the FFQ method, NFFQ-Elderly addresses a significant methodological gap in the Italian context, providing a suitable means for assessing ultra-processed food consumption in the growing population aged ≥65 years.

## Figures and Tables

**Table 1:** Participants’ characteristics.

	Whole Sample	Women	Men
Participants, *n* (%)	111 (100.0)	63 (56.8)	48 (43.2)
Age (years)	73.7 ± 5.9	72.6 ± 5.7	75.1 ± 5.9
Body mass index (kg/m^2^)	25.4 ± 3.6	25.0 ± 3.5	26.0 ± 3.8
Educational level			
Primary	24 (21.6)	17 (27.0)	7 (14.6)
Lower secondary	28 (25.2)	15 (23.8)	13 (27.1)
Upper secondary	39 (35.1)	18 (28.6)	21 (43.7)
Postsecondary	20 (18.1)	13 (20.6)	7 (14.6)
Household income (Euros/year)			
≤20,000	38 (34.2)	24 (38.1)	14 (29.2)
20,000–30,000	34 (30.6)	18 (28.6)	16 (33.3)
≥30,000	28 (25.2)	12 (19.0)	16 (33.3)
Non-responder/missing data	11 (10.0)	9 (14.3)	2 (4.2)
Number of cohabitants *			
1	21 (19.3)	14 (22.6)	7 (14.9)
2	66 (60.5)	38 (61.3)	28 (59.6)
≥3	22 (20.2)	10 (16.1)	12 (25.5)

Continuous variables are presented as mean ± SD; categorical variables as n (%). * Available for 109 participants; number of cohabitants refers to the number of individuals living in the participant’s household.

**Table 2:** Questionnaire validity in the whole sample (n = 111).

	Three-Day Food Records	NFFQ T0	r	*p*-Value	ICC	Lower Limit 95% CI	Upper Limit 95% CI
Nova groups (g/d)							
MPFs	694.3 (227.3)	832.5 (248.0)	0.57	<0.0001	0.53	0.39	0.65
PCIs	36.5 (12.5)	28.5 (11.8)	0.21	0.025	0.05	0.00	0.76
PFs	214.4 (144.1)	339.6 (176.5)	0.41	<0.0001	0.34	0.20	0.52
PCIs + PFs	250.9 (145.3)	368.1 (178.1)	0.43	<0.0001	0.37	0.23	0.54
UPFs	106.2 (80.7)	195.0 (159.8)	0.48	<0.0001	0.21	0.09	0.44
Total food intake	1051.4 (269.4)	1395.6 (355.6)	0.50	<0.0001	0.17	0.06	0.42
Nova groups (energy ratio)							
MPFs	37.2 (10.1)	31.9 (8.6)	0.48	<0.0001	0.34	0.20	0.52
PCIs	19.4 (5.9)	12.5 (5.0)	0.10	0.30	-	-	-
PFs	27.0 (12.2)	32.9 (10.7)	0.30	0.0012	0.23	0.10	0.45
PCIs + PFs	46.4 (11.0)	45.4 (10.2)	0.35	0.0002	0.37	0.23	0.54
UPFs	16.4 (10.4)	22.7 (11.5)	0.46	<0.0001	0.37	0.22	0.54
Total energy intake (kcal/d)	1577.5 (367.9)	1902.2 (512.4)	0.40	<0.0001	0.21	0.09	0.44
Nova groups (weight ratio)							
MPFs	65.7 (12.3)	60.1 (11.5)	0.60	<0.0001	0.48	0.36	0.62
PCIs	3.6 (1.3)	2.1 (0.9)	0.17	0.070	-	-	-
PFs	20.3 (11.6)	24.2 (10.0)	0.50	<0.0001	0.53	0.39	0.66
PCIs + PFs	23.9 (11.6)	26.3 (9.9)	0.52	<0.0001	0.37	0.23	0.54
UPFs	10.4 (8.1)	13.5 (8.8)	0.45	<0.0001	0.47	0.33	0.62

Data are reported as mean ± SD. Abbreviations: MPFs (unprocessed or minimally processed foods); PCIs (processed culinary ingredients); PFs (processed foods); UPFs (ultra-processed foods); CI (confidence interval); ICC (intraclass correlation coefficients); and r (Pearson correlation coefficient).

**Table 3:** Test–retest reliability (n = 110).

	NFFQ T0	NFFQ T1	r	*p*-Value	ICC	Lower Limit 95% CI	Upper Limit 95% CI
Nova groups (g/d)							
MPFs	833.1 (250.6)	861.9 (263.0)	0.75	<0.0001	0.74	0.65	0.81
PCIs	28.6 (11.8)	28.4 (14.3)	0.39	<0.0001	0.33	0.19	0.51
PFs	331.4 (175.1)	357.7 (184.7)	0.73	<0.0001	0.66	0.55	0.76
PCIs + PFs	359.9 (176.8)	386.2 (187.6)	0.73	<0.0001	0.67	0.56	0.76
UPFs	189.8 (155.2)	213.2 (140.8)	0.87	<0.0001	0.85	0.80	0.90
Total food intake	1382.8 (351.9)	1461.3 (410.6)	0.76	<0.0001	0.70	0.60	0.79
Nova groups (energy ratio)							
MPFs	32.1 (8.5)	32.1 (8.6)	0.81	<0.0001	0.80	0.73	0.86
PCIs	12.7 (5.1)	12.1 (5.5)	0.36	0.0001	0.29	0.15	0.48
PFs	32.9 (10.7)	32.8 (8.2)	0.73	<0.0001	0.69	0.58	0.79
PCIs + PFs	45.6 (10.3)	44.9 (8.8)	0.72	<0.0001	0.70	0.60	0.79
UPFs	22.3 (11.3)	23.0 (9.8)	0.82	<0.0001	0.79	0.71	0.85
Total energy intake (kcal/d)	1873.2 (502.1)	1988.7 (608.6)	0.76	<0.0001	0.74	0.64	0.81
Nova groups (weight ratio)							
MPFs	60.6 (11.4)	59.6 (10.8)	0.84	<0.0001	0.82	0.75	0.87
PCIs	2.2 (0.9)	2.0 (1.0)	0.35	0.0002	0.21	0.08	0.44
PFs	23.9 (10.0)	24.2 (8.1)	0.77	<0.0001	0.73	0.63	0.81
PCIs + PFs	26.0 (10.0)	26.2 (8.1)	0.78	<0.0001	0.70	0.60	0.79
UPFs	13.3 (8.6)	14.2 (6.8)	0.83	<0.0001	0.81	0.74	0.87

Data are reported as mean ± SD. Abbreviations: MPFs (unprocessed or minimally processed foods); PCIs (processed culinary ingredients); PFs (processed foods); UPFs (ultra-processed foods); CI (confidence interval); ICC (intraclass correlation coefficients); and r (Pearson correlation coefficient).

## Data Availability

The data presented in this study are available on request from the corresponding authors due to the ongoing nature of the study.

## Supplementary Materials

The following supporting information can be downloaded at: https://www.mdpi.com/article/10.3390/nu18081266/s1, Table S1: STROBE-nut: An extension of the STROBE statement for nutritional epidemiology; Table S2: The NFFQ-Elderly questionnaire (a) Italian version (b) English translation. Information regardin g the original FFQ, including its structure and validation, can be accessed via ref. [25] or at the following link: https://doi.org/10.1080/09637486.2021.1880552; Table S3: Questionnaire validity in women (n = 63); Table S4: Questionnaire validity in men (n = 48); Table S5: Test–retest reliability in women (n = 62); Table S6: Test–retest reliability in men (n = 48).

## Abbreviations

The following abbreviations are used in this manuscript:

NFFQ-Elderly	Nova-based Food Frequency Questionnaire for Elderly
MPFs	Unprocessed or minimally processed foods
PCIs	Processed Culinary Ingredients
PFs	Processed Foods
UPFs	Ultra-Processed Foods
FR	Food Record
ICC	Intraclass Correlation Coefficient
BMI	Body Mass Index
SD	Standard Deviation
IC	Confidence Interval
