# Supplementary material for: Reproducibility and Validity of a Nova-Based Food Frequency Questionnaire in Older Italian Adults: The NFFQ-Elderly
Source: Nutrients. 2026 Apr 16;18(8):1266. doi: 10.3390/nu18081266 (PMC13118279; doi:10.3390/nu18081266)
Supplement: Supplementary file 1 [file nutrients-18-01266-s001.zip › Tables S5 and S6(Supplementary).pdf]

Table S5. Test–retest reliability in women (n = 62).

	NFFQ T0	NFFQ T1	r	p-value	ICC	Lower Limit 95% CI	Upper Limit 95% CI
Nova groups (g/d)							
MPFs	834.7 (222.6)	863.9 (257.5)	0.73	<.0001	0.74	0.61	0.83
PCIs	31.7 (11.5)	28.3 (11.5)	0.59	<.0001	0.54	0.37	0.70
PFs	287.1 (141.5)	317.1 (156.5)	0.67	<.0001	0.65	0.50	0.78
PCIs + PFs	318.7 (140.8)	345.4 (157.6)	0.68	<.0001	0.66	0.51	0.79
UPFs	207.7 (182.1)	220.2 (152.0)	0.87	<.0001	0.87	0.79	0.92
Total food intake	1361.2 (315.6)	1429.6 (376.1)	0.72	<.0001	0.72	0.59	0.82
Nova groups (energy ratio)							
MPFs	31.8 (8.4)	32.2 (9.1)	0.81	<.0001	0.83	0.74	0.90
PCIs	14.2 (4.9)	12.4 (4.8)	0.53	<.0001	0.51	0.33	0.68
PFs	30.4 (10.7)	31.6 (8.3)	0.73	<.0001	0.69	0.55	0.80
PCIs + PFs	44.6 (10.0)	44.0 (8.4)	0.77	<.0001	0.74	0.61	0.84
UPFs	23.6 (12.1)	23.8 (10.2)	0.81	<.0001	0.78	0.66	0.86
Total energy intake (kcal/d)	1856.8 (490.6)	1952.8 (547.8)	0.73	<.0001	0.78	0.67	0.86
Nova groups (weight ratio)							
MPFs	61.9 (11.8)	61.3 (11.5)	0.86	<.0001	0.85	0.77	0.91
PCIs	2.4 (0.9)	2.1 (0.8)	0.49	<.0001	0.43	0.25	0.63
PFs	21.2 (9.0)	21.9 (7.6)	0.73	<.0001	0.71	0.57	0.82
PCIs + PFs	23.6 (8.9)	24.0 (7.5)	0.73	<.0001	0.74	0.61	0.84
UPFs	14.5 (10.0)	14.7 (7.5)	0.87	<.0001	0.83	0.74	0.89

Data are reported as mean ± SD. Abbreviations: MPFs (unprocessed or minimally processed foods); PCIs (processed culinary ingredients); PFs (processed foods); UPFs (ultra-processed foods); CI (confidence interval); ICC (intraclass correlation coefficients); r (Pearson correlation coefficients).

Table S6. Test–retest reliability in men (*n* = 48).

	NFFQ T0	NFFQ T1	r	*p*-value	ICC	Lower Limit 95% CI	Upper Limit 95% CI
Nova groups (g/d)							
MPFs	831.0 (285.1)	859.3 (272.8)	0.74	<.0001	0.74	0.60	0.85
PCIs	24.7 (11.1)	28.5 (17.4)	0.13	0.39	0.13	0.01	0.63
PFs	388.6 (198.3)	410.2 (205.7)	0.76	<.0001	0.62	0.44	0.77
PCIs + PFs	413.3 (204.0)	438.8 (210.6)	0.77	<.0001	0.63	0.45	0.78
UPFs	166.6 (108.7)	204.2 (125.9)	0.84	<.0001	0.82	0.71	0.90
Total food intake	1410.9 (395.7)	1502.2 (452.1)	0.79	<.0001	0.68	0.52	0.81
Nova groups (energy ratio)							
MPFs	32.4 (8.7)	31.9 (7.8)	0.82	<.0001	0.76	0.62	0.86
PCIs	10.8 (4.8)	11.7 (6.4)	0.12	0.40	0.01	0.00	1.00
PFs	36.2 (9.8)	34.3 (7.9)	0.72	<.0001	0.65	0.47	0.79
PCIs + PFs	47.0 (10.6)	46.0 (9.4)	0.65	<.0001	0.65	0.47	0.79
UPFs	20.6 (10.1)	22.1 (9.5)	0.81	<.0001	0.80	0.68	0.88
Total energy intake (kcal/d)	1894.4 (520.9)	2034.9 (682.3)	0.77	<.0001	0.69	0.53	0.82
Nova groups (weight ratio)							
MPFs	58.9 (10.6)	57.4 (9.4)	0.81	<.0001	0.76	0.62	0.86
PCIs	1.8 (0.8)	2.0 (1.2)	0.12	0.42	-	-	-
PFs	27.4 (10.3)	27.1 (8.0)	0.75	<.0001	0.69	0.53	0.82
PCIs + PFs	29.2 (10.5)	29.0 (8.2)	0.78	<.0001	0.65	0.47	0.79
UPFs	11.9 (6.1)	13.5 (5.8)	0.76	<.0001	0.76	0.62	0.86

Data are reported as mean ± SD. Abbreviations: MPFs (unprocessed or minimally processed foods); PCIs (processed culinary ingredients); PFs (processed foods); UPFs (ultra-processed foods); CI (confidence interval); ICC (intraclass correlation coefficients); r (Pearson correlation coefficients).
