# Supplementary material for: Reproducibility and Validity of a Nova-Based Food Frequency Questionnaire in Older Italian Adults: The NFFQ-Elderly
Source: Nutrients. 2026 Apr 16;18(8):1266. doi: 10.3390/nu18081266 (PMC13118279; doi:10.3390/nu18081266)
Supplement: Supplementary file 1 [file nutrients-18-01266-s001.zip › Table S1 STROBE-nut_checklist.pdf]

Table S1. STROBE-nut: An extension of the STROBE statement for nutritional epidemiology

Lachat C et al. (2016) STrengthening the Reporting of OBservational studies in Epidemiology – Nutritional Epidemiology (STROBE-nut): an extension of the STROBE statement. Plos Medicine 13(6) http://dx.doi.org/10.1371/journal.pmed.1002036 pdf or online version.

Item	Item nr	STROBE recommendations	Extension for Nutritional Epidemiology studies (STROBE-nut)	Reported on page #
**Title and abstract**	1	(a) Indicate the study's design with a commonly used term in the title or the abstract.	**nut-1** State the dietary/nutritional assessment method(s) used in the title, abstract, or keywords.	Page 1 (Title, abstract, keywords)
		(b) Provide in the abstract an informative and balanced summary of what was done and what was found.		Page 1 (Abstract)
**Introduction**				
Background rationale	2	Explain the scientific background and rationale for the investigation being reported.		Page 2 lines 60-89 Full Introduction
Objectives	3	State specific objectives, including any pre-specified hypotheses.		Page 2, lines 90-91
**Methods**				
Study design	4	Present key elements of study design early in the paper.		Page 3, lines 99-100; lines 103-107
Settings	5	Describe the setting, locations, and relevant dates, including periods of recruitment, exposure, follow-up, and data collection.	**nut-5** Describe any characteristics of the study settings that might affect the dietary	Page 3, lines 103-104 (Recruitment period and regions); lines 125-127 (Specific

Item	Item nr	STROBE recommendations	Extension for Nutritional Epidemiology studies (STROBE-nut)	Reported on page #
			intake or nutritional status of the participants, if applicable.	municipalities and recruitment method); lines 136-137 and page 4 figure 1 (follow-up); Page 3, lines 132-133 (data collection)
Participants	6	a) Cohort study—Give the eligibility criteria, and the sources and methods of selection of participants. Describe methods of follow-up.<br><br>Case-control study—Give the eligibility criteria, and the sources and methods of case ascertainment and control selection. Give the rationale for the choice of cases and controls.<br><br>Cross-sectional study—Give the eligibility criteria, and the sources and methods of selection of participants.	**nut-6** Report particular dietary, physiological or nutritional characteristics that were considered when selecting the target population.	Page 3, lines 104-105 (Selection sources and methods); lines 110-113 (Exclusion criteria); lines 136-138 (follow-up)
		(b) Cohort study—For matched studies, give matching criteria and number of exposed and unexposed.<br><br>Case-control study—For matched studies, give matching criteria and the number of controls per case.		Not Applicable
Variables	7	Clearly define all outcomes, exposures, predictors, potential confounders, and effect	**nut-7.1** Clearly define foods, food groups, nutrients, or other food components.	page 5, lines 204-211(potential

Item	Item nr	STROBE recommendations	Extension for Nutritional Epidemiology studies (STROBE-nut)	Reported on page #
		modifiers. Give diagnostic criteria, if applicable.	**nut-7.2** When using dietary patterns or indices, describe the methods to obtain them and their nutritional properties.	confounders and anthropometric data); **nut-7.1:** page 4, lines 158-168(Definition of FFQ items, food groups, frequency categories, portion sizes and 12-month reference period); page 5, lines 209-215 (3-day FR recording and processing coding); **nut-7.2:** pages 4/5, lines 183-193 (Nova framework classification and PCIs+PFs grouping criteria)
Data sources - measurements	8	For each variable of interest, give sources of data and details of methods of assessment (measurement).Describe comparability of assessment methods if there is more than one group.	**nut-8.1** Describe the dietary assessment method(s), e.g., portion size estimation, number of days and items recorded, how it was developed and administered, and how quality was assured. Report if and how supplement intake was assessed.	page 4, lines 158-168; page 3, lines 132- 133 and 136-137 (NFFQ-Elderly); page 5, lines 216-228 (3-day FR)
			**nut-8.2** Describe and justify food composition data used. Explain the	page 5, lines 232-235

Item	Item nr	STROBE recommendations	Extension for Nutritional Epidemiology studies (STROBE-nut)	Reported on page #
			procedure to match food composition with consumption data. Describe the use of conversion factors, if applicable.	page 5, lines 194-204
			**nut-8.3** Describe the nutrient requirements, recommendations, or dietary guidelines and the evaluation approach used to compare intake with the dietary reference values, if applicable.	Not Applicable
			**nut-8.4** When using nutritional biomarkers, additionally use the STROBE Extension for Molecular Epidemiology (STROBE-ME). Report the type of biomarkers used and their usefulness as dietary exposure markers.	Pag 5, lines 205-212
			**nut-8.5** Describe the assessment of nondietary data (e.g., nutritional status and influencing factors) and timing of the assessment of these variables in relation to dietary assessment.	Page 3, lines 136-139
			**nut-8.6** Report on the validity of the dietary or nutritional assessment methods and any internal or external validation used in the study, if applicable.	

Item	Item nr	STROBE recommendations	Extension for Nutritional Epidemiology studies (STROBE-nut)	Reported on page #
Bias	9	Describe any efforts to address potential sources of bias.	**nut-9** Report how bias in dietary or nutritional assessment was addressed, e.g., misreporting, changes in habits as a result of being measured, or data imputation from other sources	Pag 3, lines 139-142; page 5, lines 232-233; page 6, lines 241-246
Study Size	10	Explain how the study size was arrived at.		Page 3, lines 107-109
Quantitative variables	11	Explain how quantitative variables were handled in the analyses. If applicable, describe which groupings were chosen and why.	**nut-11** Explain categorization of dietary/nutritional data (e.g., use of N-tiles and handling of nonconsumers) and the choice of reference category, if applicable.	Page 4, lines 158-168; page 5, lines 194-204; page 5, lines 222-228
Statistical Methods	12	(a) Describe all statistical methods, including those used to control for confounding<br>(b) Describe any methods used to examine subgroups and interactions.<br>(c) Explain how missing data were addressed.<br>(d) Cohort study—If applicable, explain how loss to follow-up was addressed.<br>Case-control study—If applicable, explain how matching of cases and controls was addressed.	**nut-12.1** Describe any statistical method used to combine dietary or nutritional data, if applicable.<br>**nut-12.2** Describe and justify the method for energy adjustments, intake modeling, and use of weighting factors, if applicable.<br>**nut-12.3** Report any adjustments for measurement error, i.e,. from a validity or calibration study.	Page 6, lines 259-275; Page 6, lines 271-273<br>12(c) (Missing/Excluded Data) page 6, lines 241-246<br>Page 6, lines 271-275<br>Page 6, lines 268-271

Item	Item nr	STROBE recommendations	Extension for Nutritional Epidemiology studies (STROBE-nut)	Reported on page #
		Cross-sectional study—If applicable, describe analytical methods taking account of sampling strategy.<br><br>(e) Describe any sensitivity analyses.		
**Results**				
Participants	13	(a) Report the numbers of individuals at each stage of the study—e.g., numbers potentially eligible, examined for eligibility, confirmed eligible, included in the study, completing follow-up, and analyzed.<br><br>(b) Give reasons for non-participation at each stage.<br><br>(c) Consider use of a flow diagram.	**nut-13** Report the number of individuals excluded based on missing, incomplete or implausible dietary/nutritional data.	Page 6, lines 279-283(Initial recruitment and validation sample n=111); Page 6, lines 288–290(Subsample for test-retest n=110);<br><br>Page 6, lines 279-283(Detailed reasons for exclusion: 2 incomplete FR, 4 implausible energy intakes); 13 c: Not Applicable
Descriptive data	14	(a) Give characteristics of study participants (e.g., demographic, clinical, social) and information on exposures and potential confounders<br><br>(b) Indicate the number of participants with missing data for each variable of interest	**nut-14** Give the distribution of participant characteristics across the exposure variables if applicable. Specify if food consumption of total population or consumers only were used to obtain results.	Page 6, lines 285-288 and Table 1

Item	Item nr	STROBE recommendations	Extension for Nutritional Epidemiology studies (STROBE-nut)	Reported on page #
		(c) Cohort study—Summarize follow-up time (e.g., average and total amount)		(b): Table 1 (Row "Nonresponder/missing data" for income and footnote * for number of cohabitants). C: Not Applicable
Outcome data	15	Cohort study—Report numbers of outcome events or summary measures over time.<br><br>Case-control study—Report numbers in each exposure category, or summary measures of exposure.<br><br>Cross-sectional study—Report numbers of outcome events or summary measures.		Page 8, lines 310-313; Table 2 (Summary measures for relative validity); Table 3 (Summary measures for test–retest reliability)
Main results	16	(a) Give unadjusted estimates and, if applicable, confounder-adjusted estimates and their precision (e.g., 95% confidence interval).<br><br>Make clear which confounders were adjusted for and why they were included.<br><br>(b) Report category boundaries when continuous variables were categorized.	**nut-16** Specify if nutrient intakes are reported with or without inclusion of dietary supplement intake, if applicable.	Page 8, lines 314-326 (Absolute and relative validity coefficients); page 8, lines 327-332 (Bland-Altman analysis and systematic bias);page 10, lines 364-370(Test–retest reliability coefficients); Table 2, Table 3, and Figure 2.

Item	Item nr	STROBE recommendations	Extension for Nutritional Epidemiology studies (STROBE-nut)	Reported on page #
		(c) If relevant, consider translating estimates of relative risk into absolute risk for a meaningful time period.		(b) and (c) Not Applicable
Other analyses	17	Report other analyses done—e.g., analyses of subgroups and interactions and sensitivity analyses.	**nut-17** Report any sensitivity analysis (e.g., exclusion of misreporters or outliers) and data imputation, if applicable.	Page 8, lines 333-335 (Sex-stratified validity); Page 10, lines 371-373 (Sex-stratified reliability); Supplementary Tables S3-S6; page 6, lines 241-246
**Discussion**				
Key results	18	Summarize key results with reference to study objectives.		Page 12, lines 383-386
Limitation	19	Discuss limitations of the study, taking into account sources of potential bias or imprecision. Discuss both direction and magnitude of any potential bias.	**nut-19** Describe the main limitations of the data sources and assessment methods used and implications for the interpretation of the findings.	Pages 13/14, lines 463-492

**Item**	**Item nr**	**STROBE recommendations**	**Extension for Nutritional Epidemiology studies (STROBE-nut)**	**Reported on page #**
Interpretation	20	Give a cautious overall interpretation of results considering objectives, limitations, multiplicity of analyses, results from similar studies, and other relevant evidence.	**nut-20** Report the nutritional relevance of the findings, given the complexity of diet or nutrition as an exposure.	Page 12, lines 415-422; lines 423-427; page 13, line 441-445
Generalizability	21	Discuss the generalizability (external validity) of the study results.		Page 13, lines 480-484
**Other information**				
Funding	22	Give the source of funding and the role of the funders for the present study and, if applicable, for the original study on which the present article is based.		Page 14, Funding section
Ethics			**nut-22.1** Describe the procedure for consent and study approval from ethics committee(s).	Page 3, lines 114-119
Supplementary material			**nut-22.2** Provide data collection tools and data as online material or explain how they can be accessed.	Page 14, Supplementary Materials section
