# Supplementary material for: Reproducibility and Validity of a Nova-Based Food Frequency Questionnaire in Older Italian Adults: The NFFQ-Elderly
Source: Nutrients. 2026 Apr 16;18(8):1266. doi: 10.3390/nu18081266 (PMC13118279; doi:10.3390/nu18081266)
Supplement: Supplementary file 1 [file nutrients-18-01266-s001.zip › Table S2 a NFFQ-Elderly.pdf]

Gentile partecipante,

Ti chiediamo pochi minuti del tuo tempo per compilare questo questionario che ci aiuterà a capire meglio il consumo di alimenti ultra-processati nella popolazione italiana.

La compilazione di questo questionario rientra nell'ambito del progetto **NUTRAGE "NUTRIZIONE, ALIMENTAZIONE & INVECCHIAMENTO ATTIVO". NUTRAGE è un progetto multidisciplinare, promosso e sviluppato dal Consiglio Nazionale delle Ricerche e finanziato dai fondi FOE 2021** che ha l'obiettivo di individuare i migliori regimi dietetici in grado di prevenire o ritardare l'insorgenza delle principali patologie neurodegenerative e metaboliche legate all'invecchiamento e **formulare programmi specifici di nutrizione personalizzata** per la terza età.

Compilando il questionario, ti chiediamo di considerare le tue abitudini alimentari nell'arco dell'**ultimo anno.**

Per ogni alimento, considera sia la frequenza con cui lo consumi che le quantità medie consumate ogni qualvolta lo hai mangiato.

Ecco un esempio di come compilare il questionario:

- ✓ Se mangi un alimento "Mai o meno di una volta al mese", puoi non indicare la quantità;
- ✓ Se consumi un alimento più di una volta al mese, contrassegna anche la casella relativa alla quantità consumata in termini di porzione media in ogni occasione di consumo;
- ✓ Se consumi un alimento ogni giorno, indica con un numero anche le volte al giorno che lo ha consumato.

Se usi gli alimenti per preparare ricette specifiche (che non trovi nell'elenco) considera i singoli alimenti nelle relative sezioni ed indica la frequenza e le quantità consumate. Nel caso in cui non trovi nella lista alcuni alimenti che consumi o utilizzi abitualmente, nell'ultima pagina c'è una sezione dove potrai inserirli.

Se hai cambiato la tua dieta per motivi di salute o personali per brevi periodi, ricorda di compilare il questionario riferendosi alla tua abituale alimentazione

FRUTTA E FRUTTA SECCA

	**Frequenza di consumo** *Per favore indica una sola risposta per ciascun alimento*										**Quantità** *Per favore indica una sola risposta per ciascun alimento*						
**Frutta e frutta secca**	Mai o meno di 1 volta al mese	1-3 volte al mese	1 volta a settimana	2 volta a settimana	3 volte a settimana	4 volte a settimana	5 volte a settimana	6 volte a settimana	Tutti i giorni	Se tutti i giorni quante volte al giorno	**Porzione di riferimento**	**La sua porzione in ogni occasione d'uso**					
**Frutta** (fresca, tagliata, frullata,surgelata)	X										**150g** (es. 1 frutto medio: mela, pera, arancia 2 frutti piccoli: albicocche, mandarini ecc.)	0,5	1,0	1,5	2,0	2,5	3,0
**Succhi 100% frutta** (freschi o pastorizzati, senza aggiunta di zuccheri o altri ingredienti)				X							**200ml** (1 bicchiere medio da acqua)	0,5	1,0	1,5	X 2,0	2,5	3,0
**Succhi di frutta/nettari** (con aggiunta di zuccheri o altri ingredienti)									X	3	**200ml** (1 bicchiere medio da acqua)	X 0,5	1,0	1,5	2,0	2,5	3,0

GRAZIE PER LA COLLABORAZIONE!!

FRUTTA E FRUTTA SECCA

	**Frequenza di consumo**<br>*Per favore indica una sola risposta per ciascun alimento*										**Quantità**<br>*Per favore indica una sola risposta per ciascun alimento*						
**Frutta e frutta secca**	Mai o meno di 1 volta al mese	1-3 volte al mese	1 volta a settimana	2 volta a settimana	3 volte a settimana	4 volte a settimana	5 volte a settimana	6 volte a settimana	Tutti i giorni	Se tutti i giorni quante volte al giorno	**Porzione di riferimento**	**La sua porzione in ogni occasione d'uso**					
**Frutta** (fresca, tagliata, frullata,surgelata)											**150g** (es. 1 frutto medio: mela, pera, arancia 2 frutti piccoli: albicocche, mandarini ecc.)	0,5	1,0	1,5	2,0	2,5	3,0
**Succhi 100% frutta** (freschi o pastorizzati, senza aggiunta di zuccheri o altri ingredienti)											**200ml** (1 bicchiere medio)	0,5	1,0	1,5	2,0	2,5	3,0
**Succhi di frutta/nettari e bevande a base di frutta** (con aggiunta di zuccheri o altri ingredienti)											**200ml** (1 bicchiere medio)	0,5	1,0	1,5	2,0	2,5	3,0
**Frutta sciroppata**											**150g** (es. 3 mezze pesche sciroppate)	0,5	1,0	1,5	2,0	2,5	3,0
**Frutta essiccata** (es. albicocche, prugne, fichi,datteri)											**30g** (es. 3 fichi secchi/datteri, 2 cucchiai rasi di uvetta)	0,5	1,0	1,5	2,0	2,5	3,0
**Frutta secca e semi (**senza aggiunta di sale, zucchero o altri ingredienti)											**30g** (es. 7-8 noci, 15-20 mandorle/nocciole, 3 cucchiai di semi di girasole ecc.)	0,5	1,0	1,5	2,0	2,5	3,0
**Frutta secca e semi** (con aggiunta di sale, zucchero o altri ingredienti)											**30g** (es. 7-8 noci, 15-20 mandorle/nocciole, 3 cucchiai rasi di semi di girasole ecc.)	0,5	1,0	1,5	2,0	2,5	3,0
**Omogeneizzato alla frutta fatto in casa**											**80g** (es. 1 vasetto)	0,5	1,0	1,5	2,0	2,5	3,0
**Omogeneizzato alla frutta confezionato**											**80g** (es. 1 vasetto)	0,5	1,0	1,5	2,0	2,5	3,0
**Olive da tavola**											**35g** (es. 5 olive)	0,5	1,0	1,5	2,0	2,5	3,0

VERDURE E LEGUMI

	**Frequenza di consumo** *Per favore indica una sola risposta per ciascun alimento*										**Quantità** *Per favore indica una sola risposta per ciascun alimento*						
**Verdura e Legumi**	Mai o meno di 1 volta al mese	1-3 volte al mese	1 volta a settimana	2 volta a settimana	3 volte a settimana	4 volte a settimana	5 volte a settimana	6 volte a settimana	Tutti i giorni	Se tutti i giorni quante volte al giorno	**Porzione di riferimento**	**La sua porzione in ogni occasione d'uso**					
**Verdure e ortaggi freschi o surgelati al naturale** (crudi o cotti)										________	**200g** 2-3 pomodori, 1 finocchio, ½ piatto di spinaci/**80g** insalata es. 1 scodella/ciotola grande da 500ml	0,5	1,0	1,5	2,0	2,5	3,0
**Verdure e ortaggi confezionati pronti all'uso/da cuocere**										________	**200g** (½ piatto di spinaci con formaggio)	0,5	1,0	1,5	2,0	2,5	3,0
**Succhi 100% verdura** (freschi o pastorizzati senza aggiunta di zuccheri oaltri ingredienti)										________	**200ml** (1 bicchiere medio)	0,5	1,0	1,5	2,0	2,5	3,0
**Verdure in lattina o in bottiglia** (es. salsa di pomodoro, sottaceti, sott'olio)										________	**150g (**es. 1 bicchiere piccolo di salsa di pomodoro,10 cetriolini medi ecc.) / **50g (es.**2-3 carciofini sott'olio)	0,5	1,0	1,5	2,0	2,5	3,0
**Legumi freschi o secchi** (es. fagioli, piselli, lenticchie ecc.)										________	**150g** (½ piatto per freschi) /**50g** (3-4 cucchiai per secchi)	0,5	1,0	1,5	2,0	2,5	3,0
**Legumi in lattina** (con aggiunta di sale o altri ingredienti)										________	**150g** (es. 1 scatola piccola)	0,5	1,0	1,5	2,0	2,5	3,0
**Legumi in lattina confezionati pronti all'uso/da cuocere** (es. alla cacciatora, alla messicana, frijoles)										________	**200g** (es. 1 scatola piccola)	0,5	1,0	1,5	2,0	2,5	3,0
**Omogeneizzato di verdure fatto in casa**										________	**80g** (es. 1 vasetto)	0,5	1,0	1,5	2,0	2,5	3,0
**Omogeneizzato di verdure confezionato**										________	**80g** (es. 1 vasetto)	0,5	1,0	1,5	2,0	2,5	3,0

CEREALI E TUBERI

	**Frequenza di consumo**<br>*Per favore indica una sola risposta per ciascun alimento*										**Quantità**<br>*Per favore indica una sola risposta per ciascun alimento*						
**Cereali e Tuberi**	Mai o meno di 1 volta al mese	1-3 volte al mese	1 volta a settimana	2 volta a settimana	3 volte a settimana	4 volte a settimana	5 volte a settimana	6 volte a settimana	Tutti i giorni	Se tutti i giorni quante volte al giorno	**Porzione di riferimento**	**La sua porzione in ogni occasione d'uso**					
**Cereali in chicco** (es. riso, farro, orzo, avena, frumento ecc.)										______	**80g** (1 piatto medio)	0,5	1,0	1,5	2,0	2,5	3,0
**Pasta secca o fresca, polenta, cous cous, semolino**										______	**80g secchi / 125g freschi (**1 piatto medio)	0,5	1,0	1,5	2,0	2,5	3,0
**Pasta ripiena tipo ravioli, lasagne, gnocchi di patate fatti in casa o artigianali**										______	**200g (**circa 1 piatto medio)	0,5	1,0	1,5	2,0	2,5	3,0
**Pasta ripiena tipo ravioli, lasagne, gnocchi di patate o risotti confezionati pronti all'uso/da cuocere**										______	**200g (**circa 1 piatto medio)	0,5	1,0	1,5	2,0	2,5	3,0
**Noodles, riso o zuppe istantanee**										______	**90g** (1 porzione)	0,5	1,0	1,5	2,0	2,5	3,0
**Pane fresco e panini (**fatti in casa o artigianali)										______	**50g** (es. 1 fetta media di pagnotta, 1 panino piccolo)	0,5	1,0	1,5	2,0	2,5	3,0
**Pane e panini confezionati (**es. pancarrè)										______	**50g** (es. 2-3 fette, 1 panino piccolo)	0,5	1,0	1,5	2,0	2,5	3,0

CEREALI E TUBERI

	**Frequenza di consumo**<br>*Per favore indica una sola risposta per ciascun alimento*										**Quantità**<br>*Per favore indica una sola risposta per ciascun alimento*						
**Cereali e Tuberi**	Mai o meno di 1 volta al mese	1-3 volte al mese	1 volta a settimana	2 volta a settimana	3 volte a settimana	4 volte a settimana	5 volte a settimana	6 volte a settimana	Tutti i giorni	Se tutti i giorni quante volte al giorno	**Porzione di riferimento**	**La sua porzione in ogni occasione d'uso**					
**Sostituti del pane** (es. crakers, taralli, grissini, friselle, fette biscottate)	○	○	○	○	○	○	○	○	○	________	**30g** (es.1 pacchetto di cracker, 3-4 tarallini, 1 frisella, 3-4 fette biscottate)	○ 0,5	○ 1,0	○ 1,5	○ 2,0	○ 2,5	○ 3,0
**Tramezzini, sandwich confezionati**	○	○	○	○	○	○	○	○	○	________	**80g (**es. 1 tramezzino)	○ 0,5	○ 1,0	○ 1,5	○ 2,0	○ 2,5	○ 3,0
**Pizza, focaccia fatta in casa o artigianale**	○	○	○	○	○	○	○	○	○	________	**350g** (1 pizza)	○ 0,5	○ 1,0	○ 1,5	○ 2,0	○ 2,5	○ 3,0
**Pizza, focaccia, confezionati pronti all'uso/da cuocere tipo surgelata)**	○	○	○	○	○	○	○	○	○	________	**350g** (1 pizza)	○ 0,5	○ 1,0	○ 1,5	○ 2,0	○ 2,5	○ 3,0
**Torte salate, rustici fatte in casa artigianali**	○	○	○	○	○	○	○	○	○	________	**150g** (1 fetta media)	○ 0,5	○ 1,0	○ 1,5	○ 2,0	○ 2,5	○ 3,0
**Torte salate, rustici confezionate**	○	○	○	○	○	○	○	○	○	________	**150g** (1 fetta media)	○ 0,5	○ 1,0	○ 1,5	○ 2,0	○ 2,5	○ 3,0
**Cereali da colazione in fiocchi senza zuccheri aggiunti o muesli con frutta secca o essiccata senza altri ingredienti**	○	○	○	○	○	○	○	○	○	________	**30g** (es. 6-8 cucchiai di fiocchi di mais, 5-6 cucchiai di altri cereali in fiocchi più pesanti,3 cucchiai di muesli)	○ 0,5	○ 1,0	○ 1,5	○ 2,0	○ 2,5	○ 3,0
**Cereali per la colazione, muesli o granola con zuccheri o altri ingredienti aggiunti,e barrette di cereali**	○	○	○	○	○	○	○	○	○	________	**30g** (6-8 cucchiai di fiocchi di mais, 5-6 cucchiai di altri cereali in fiocchi più pesanti, 3 cucchiai di muesli	○ 0,5	○ 1,0	○ 1,5	○ 2,0	○ 2,5	○ 3,0
**Patate, crocchette di patate, gateau e simili o fatti in casa o artigianali)**	○	○	○	○	○	○	○	○	○	________	**200g** (2 patate piccole)	○ 0,5	○ 1,0	○ 1,5	○ 2,0	○ 2,5	○ 3,0
**Patate, crocchette di patate e simili confezionati pronti all'uso/da cuocere**	○	○	○	○	○	○	○	○	○	________	**150g** (1 piatto medio)	○ 0,5	○ 1,0	○ 1,5	○ 2,0	○ 2,5	○ 3,0

CARNE O PESCE

	**Frequenza di consumo**<br>*Per favore indica una sola risposta per ciascun alimento*										**Quantità**<br>*Per favore indica una sola risposta per ciascun alimento*						
**Carne e Pesce**	Mai o meno di 1 volta al mese	1-3 volte al mese	1 volta a settimana	2 volta a settimana	3 volte a settimana	4 volte a settimana	5 volte a settimana	6 volte a settimana	Tutti i giorni	Se tutti i giorni quante volte al giorno	**Porzione di riferimento**	**La sua porzione in ogni occasione d'uso**					
**Carne e pollame** (es. bistecche, filetti o altri tagli) fresca o surgelata senza altri ingredienti	○	○	○	○	○	○	○	○	○	______	**100g** (es. 1 fettina di carne, 1 piccola coscia di pollo)	○ 0,5	○ 1,0	○ 1,5	○ 2,0	○ 2,5	○ 3,0
**Affettati, salumi e carne affumicata** (inclusi se usati per preparare panini)	○	○	○	○	○	○	○	○	○	______	**50g** (es. 3-4 fette medie di prosciutto, 5-6 fette medie di salame o di bresaola	○ 0,5	○ 1,0	○ 1,5	○ 2,0	○ 2,5	○ 3,0
**Cotolette, salcicce, hamburger fatte in casa**	○	○	○	○	○	○	○	○	○	______	**100g** (es. 1 salsiccia, 1 hamburger)	○ 0,5	○ 1,0	○ 1,5	○ 2,0	○ 2,5	○ 3,0
**Cotolette, nuggets e bastoncini di carne confezionati pronti all'uso/da cuocere**	○	○	○	○	○	○	○	○	○	______	**100g (**es 1 cotoletta 5-6 nuggets di pollo,)	○ 0,5	○ 1,0	○ 1,5	○ 2,0	○ 2,5	○ 3,0
**Salsicce, wurstel, hamburger, carne in scatola e altri prodotti a base di carne confezionati pronti all'uso/da cuocere**	○	○	○	○	○	○	○	○	○	______	**100g** (es. 1 salsiccia, 1 hamburger, 4 wurstel piccoli)	○ 0,5	○ 1,0	○ 1,5	○ 2,0	○ 2,5	○ 3,0
**Pesce, crostacei e molluschi** (es. filetti, tranci o altri tagli) freschi o surgelati	○	○	○	○	○	○	○	○	○	______	**150g** (es. 1 piccolo pesce, 1 filetto medio, 3 gamberoni, 25 cozze)	○ 0,5	○ 1,0	○ 1,5	○ 2,0	○ 2,5	○ 3,0
**Pesce affumicato, essiccato o in salamoia**	○	○	○	○	○	○	○	○	○	______	**50g** (4-5 fette sottili di salmone affumicato, ½ filetto di baccalà)	○ 0,5	○ 1,0	○ 1,5	○ 2,0	○ 2,5	○ 3,0
**Pesce in lattina o in vetro al naturale** (con o senza conservanti)	○	○	○	○	○	○	○	○	○	______	**50g** (es. 1 scatoletta piccola di tonno)	○ 0,5	○ 1,0	○ 1,5	○ 2,0	○ 2,5	○ 3,0
**Pesce in lattina o in vetro in olio** (con o senza conservanti)	○	○	○	○	○	○	○	○	○	______	**50g** (es. 1 scatoletta piccola di tonno)	○ 0,5	○ 1,0	○ 1,5	○ 2,0	○ 2,5	○ 3,0
**Cotolette, bastoncini di pesce fatti in casa**	○	○	○	○	○	○	○	○	○	______	**100g** (es. 1 cotoletta, 4 bastoncini di pesce)	○ 0,5	○ 1,0	○ 1,5	○ 2,0	○ 2,5	○ 3,0
**Cotolette, bastoncini di pesce, nuggets confezionati pronti all'uso/da cuocere**	○	○	○	○	○	○	○	○	○	______	**100g** (es. 4 bastoncini di pesce)	○ 0,5	○ 1,0	○ 1,5	○ 2,0	○ 2,5	○ 3,0

	Frequenza di consumo<br>Per favore indica una sola risposta per ciascun alimento										Quantità<br>Per favore indica una sola risposta per ciascun alimento						
**Carne e Pesce**	Mai o meno di 1 volta al mese	1-3 volte al mese	1 volta a settimana	2 volta a settimana	3 volte a settimana	4 volte a settimana	5 volte a settimana	6 volte a settimana	Tutti i giorni	Se tutti i giorni quante volte al giorno	**Porzione di riferimento**	**La sua porzione in ogni occasione d'uso**					
**Piatti pronti a base di pesce o carne confezionati** (es. cozze cotte al pomodoro, tranci di salmone marinati, insalata di mare, pollo alla diavola)											1 porzione	0,5	1,0	1,5	2,0	2,5	3,0

LATTE, LATTICINI E UOVA

	**Frequenza di consumo**<br>*Per favore indica una sola risposta per ciascun alimento*										**Quantità**<br>*Per favore indica una sola risposta per ciascun alimento*						
**Latte, latticini e uova**	Mai o meno di 1 volta al mese	1-3 volte al mese	1 volta a settimana	2 volta a settimana	3 volte a settimana	4 volte a settimana	5 volte a settimana	6 volte a settimana	Tutti i giorni	Se tutti i giorni quante volte al giorno	**Porzione di riferimento**	**La sua porzione in ogni occasione d'uso**					
**Latte di mucca, di pecora o di capra** (pastorizzato o a lunga conservazione)											**125ml** (es. 1 bicchiere piccolo, ½ tazza media)	0,5	1,0	1,5	2,0	2,5	3,0
**Latte vegetale** (es. di soia, di mandorla, di avena, di nocciola)											**125ml** (es. 1 bicchiere piccolo, ½ tazza media)	0,5	1,0	1,5	2,0	2,5	3,0
**Yogurt bianco** (senza zuccheri aggiunti)											**125ml** (es. 1 vasetto)	0,5	1,0	1,5	2,0	2,5	3,0
**Yogurt alla frutta o altri gusti (es. caffè, vaniglia) e bevande a base di latte tipo fermentato, kefir**											**125ml** (es. 1 vasetto, 1 bottiglietta)	0,5	1,0	1,5	2,0	2,5	3,0
**Ricotta di vacca, pecora**											**100g** (1 porzione)	0,5	1,0	1,5	2,0	2,5	3,0
**Formaggi molli** (inclusi se usati per preparare panini)											**100g** (es. 1 mozzarella piccola)	0,5	1,0	1,5	2,0	2,5	3,0
**Formaggi duri** (inclusi se usati per preparare panini)											**50g** (es. 2 fette di pecorino)	0,5	1,0	1,5	2,0	2,5	3,0
**Formaggi fusi, spalmabili** (inclusi se usati per preparare panini)											**30g** (es. 1 sottiletta, 1 formaggino) / 80g spalmabile	0,5	1,0	1,5	2,0	2,5	3,0
**Formaggio per condimento** (es. grana, parmigiano, pecorino)											**10g** (1 cucchiaio)	0,5	1,0	1,5	2,0	2,5	3,0

	Frequenza di consumo<br>*Per favore indica una sola risposta per ciascun alimento*										**Quantità**<br>*Per favore indica una sola risposta per ciascun alimento*						
**Latte, latticini e uova**	Mai o meno di 1 volta al mese	1-3 volte al mese	1 volta a settimana	2 volta a settimana	3 volte a settimana	4 volte a settimana	5 volte a settimana	6 volte a settimana	Tutti i giorni	Se tutti i giorni quante volte al giorno	**Porzione di riferimento**	**La sua porzione in ogni occasione d'uso**					
**Panna**											**10ml** (1 cucchiaio)	0,5	1,0	1,5	2,0	2,5	3,0
**Uova** (incluse se usate per frittate o per farcire panini)											**50g** (1 uovo)	0,5	1,0	1,5	2,0	2,5	3,0

OLI, GRASSI E CONDIMENTI

	**Frequenza di consumo**<br>*Per favore indica una sola risposta per ciascun alimento*										**Quantità**<br>*Per favore indica una sola risposta per ciascun alimento*						
**Oli, grassi e condimenti**	Mai o meno di 1 volta al mese	1-3 volte al mese	1 volta a settimana	2 volta a settimana	3 volte a settimana	4 volte a settimana	5 volte a settimana	6 volte a settimana	Tutti i giorni	Se tutti i giorni quante volte al giorno	**Porzione di riferimento**	**La sua porzione in ogni occasione d'uso**					
**Olio di oliva, olio extra vergine di oliva**											**10ml** (1 cucchiaio)	0,5	1,0	1,5	2,0	2,5	3,0
**Olio di semi**											**10ml** (1 cucchiaio)	0,5	1,0	1,5	2,0	2,5	3,0
**Burro,** (incluso il burro per la pasta in bianco), lardo, strutto, sugna											**10g** (½ noce, 1 confezione alberghiera)	0,5	1,0	1,5	2,0	2,5	3,0
**Margarina**											**10g** (½ noce, 1 confezione alberghiera)	0,5	1,0	1,5	2,0	2,5	3,0
**Salse pronte** (es. maionese, ketchup)											**18g** (1 cucchiaio colmo)	0,5	1,0	1,5	2,0	2,5	3,0
**Sughi fatti in casa** (es. pesto, ragù)											**50g (**1 porzione)	0,5	1,0	1,5	2,0	2,5	3,0
**Sughi pronti** (es. pesto, ragù)											**50g (**1 porzione)	0,5	1,0	1,5	2,0	2,5	3,0

DOLCI E DOLCIFICANTI

	**Frequenza di consumo** *Per favore indica una sola risposta per ciascun alimento*										**Quantità** *Per favore indica una sola risposta per ciascun alimento*						
**Dolci e dolcificanti**	Mai o meno di 1 volta al mese	1-3 volte al mese	1 volta a settimana	2 volta a settimana	3 volte a settimana	4 volte a settimana	5 volte a settimana	6 volte a settimana	Tutti i giorni	Se tutti i giorni quante volte al giorno	**Porzione di riferimento**	**La sua porzione in ogni occasione d'uso**					
**Biscotti fatti in casa o artigianali**	○	○	○	○	○	○	○	○	○	______	**30g** (es. 2-3 frollini, 4-5 biscotti secchi)	○ 0,5	○ 1,0	○ 1,5	○ 2,0	○ 2,5	○ 3,0
**Biscotti confezionati**	○	○	○	○	○	○	○	○	○	______	**30g** (es. 2-3 frollini, 4-5 biscotti secchi)	○ 0,5	○ 1,0	○ 1,5	○ 2,0	○ 2,5	○ 3,0
**Torte, dolci al cucchiaio fatte in casa, artigianali**	○	○	○	○	○	○	○	○	○	______	**100g** (1 fetta piccola, 1 coppetta)	○ 0,5	○ 1,0	○ 1,5	○ 2,0	○ 2,5	○ 3,0
**Torte, dolci al cucchiaio, preparati confezionati per torte o dessert**	○	○	○	○	○	○	○	○	○	______	**100g** (1 fetta piccola, 1 coppetta)	○ 0,5	○ 1,0	○ 1,5	○ 2,0	○ 2,5	○ 3,0
**Croissant, merendine confezionate**	○	○	○	○	○	○	○	○	○	______	**50g** (1 pezzo)	○ 0,5	○ 1,0	○ 1,5	○ 2,0	○ 2,5	○ 3,0
**Gelati freschi o artigianali**	○	○	○	○	○	○	○	○	○	______	**100g (**1 porzione)	○ 0,5	○ 1,0	○ 1,5	○ 2,0	○ 2,5	○ 3,0
**Gelati confezionati** (es. cono, stecco, vaschetta)	○	○	○	○	○	○	○	○	○	______	**70g** (1 cono,1 stecco,1 coppetta)	○ 0,5	○ 1,0	○ 1,5	○ 2,0	○ 2,5	○ 3,0
**Cioccolato**	○	○	○	○	○	○	○	○	○	______	**30g** (es. 2-3 cioccolattini)	○ 0,5	○ 1,0	○ 1,5	○ 2,0	○ 2,5	○ 3,0
**Caramelle**	○	○	○	○	○	○	○	○	○	______	**10g** (es. 2-3 caramelle)	○ 0,5	○ 1,0	○ 1,5	○ 2,0	○ 2,5	○ 3,0
**Creme spalmabili** (es. di nocciole, di pistacchi)	○	○	○	○	○	○	○	○	○	______	**15g** (1 cucchiaio)	○ 0,5	○ 1,0	○ 1,5	○ 2,0	○ 2,5	○ 3,0
**Marmellate e confetture fatte in casa**	○	○	○	○	○	○	○	○	○	______	**20g** (2 cucchiai)	○ 0,5	○ 1,0	○ 1,5	○ 2,0	○ 2,5	○ 3,0
**Marmellate e confetture confezionate**	○	○	○	○	○	○	○	○	○	______	**20g** (2 cucchia)	○ 0,5	○ 1,0	○ 1,5	○ 2,0	○ 2,5	○ 3,0
**Zucchero, fruttosio, melassa**	○	○	○	○	○	○	○	○	○	______	**5g** (1 cucchiaino)	○ 0,5	○ 1,0	○ 1,5	○ 2,0	○ 2,5	○ 3,0

	Frequenza di consumo<br>*Per favore indica una sola risposta per ciascun alimento*										Quantità<br>*Per favore indica una sola risposta per ciascun alimento*						
**Dolci e dolcificanti**	Mai o meno di 1 volta al mese	1-3 volte al mese	1 volta a settimana	2 volta a settimana	3 volte a settimana	4 volte a settimana	5 volte a settimana	6 volte a settimana	Tutti i giorni	Se tutti i giorni quante volte al giorno	**Porzione di riferimento**	**La sua porzione in ogni occasione d'uso**					
**Miele, sciroppo d'acero**	◯	◯	◯	◯	◯	◯	◯	◯	◯	________	**10g (**1 cucchiaino)	◯ 0,5	◯ 1,0	◯ 1,5	◯ 2,0	◯ 2,5	◯ 3,0
**Dolcificanti** (es. stevia aspartame, saccarina)	◯	◯	◯	◯	◯	◯	◯	◯	◯	________	**1 bustina,1 compressa, 2 gocce**	◯ 0,5	◯ 1,0	◯ 1,5	◯ 2,0	◯ 2,5	◯ 3,0

BEVANDE

	Frequenza di consumo<br>*Per favore indica una sola risposta per ciascun alimento*										Quantità<br>*Per favore indica una sola risposta per ciascun alimento*						
**Bevande**	Mai o meno di 1 volta al mese	1-3 volte al mese	1 volta a settimana	2 volta a settimana	3 volte a settimana	4 volte a settimana	5 volte a settimana	6 volte a settimana	Tutti i giorni	Se tutti i giorni quante volte al giorno	**Porzione di riferimento**	**La sua porzione in ogni occasione d'uso**					
**Tè, infusi o tisane**	◯	◯	◯	◯	◯	◯	◯	◯	◯	________	**125ml** (1 tazza piccola)	◯ 0,5	◯ 1,0	◯ 1,5	◯ 2,0	◯ 2,5	◯ 3,0
**Caffè**	◯	◯	◯	◯	◯	◯	◯	◯	◯	________	**30 ml (**1 tazzina)	◯ 0,5	◯ 1,0	◯ 1,5	◯ 2,0	◯ 2,5	◯ 3,0
**Soft drinks** (es. tè freddo, aranciata, cola, bevande sportive, analcolici per aperitivo)	◯	◯	◯	◯	◯	◯	◯	◯	◯	________	**330ml** (1 lattina)	◯ 0,5	◯ 1,0	◯ 1,5	◯ 2,0	◯ 2,5	◯ 3,0
**Bevande energetiche**	◯	◯	◯	◯	◯	◯	◯	◯	◯	________	**250ml** (1lattina)	◯ 0,5	◯ 1,0	◯ 1,5	◯ 2,0	◯ 2,5	◯ 3,0
**Bevande a base di cacao** (es. cioccolata calda)	◯	◯	◯	◯	◯	◯	◯	◯	◯	________	**125ml** (1 bicchiere piccolo)	◯ 0,5	◯ 1,0	◯ 1,5	◯ 2,0	◯ 2,5	◯ 3,0
**Vino**	◯	◯	◯	◯	◯	◯	◯	◯	◯	________	**125ml** (1 bicchiere piccolo)	◯ 0,5	◯ 1,0	◯ 1,5	◯ 2,0	◯ 2,5	◯ 3,0
**Birra**	◯	◯	◯	◯	◯	◯	◯	◯	◯	________	**30ml (**1 lattina)	◯ 0,5	◯ 1,0	◯ 1,5	◯ 2,0	◯ 2,5	◯ 3,0

**Altre bevande alcoliche** (es. amari, rum, gin, grappa)	○	○	○	○	○	○	○	○	○	___	**40ml**	○ 0,5	○ 1,0	○ 1,5	○ 2,0	○ 2,5	○ 3,0

ALTRO

	**Frequenza di consumo** *Per favore indica una sola risposta per ciascun alimento*										**Quantità** *Per favore indica una sola risposta per ciascun alimento*						
**Altro**	Mai o meno di 1 volta al mese	1-3 volte al mese	1 volta a settimana	2 volta a settimana	3 volte a settimana	4 volte a settimana	5 volte a settimana	6 volte a settimana	Tutti i giorni	Se tutti i giorni quante volte al giorno	**Porzione di riferimento**	**La sua porzione in ogni occasione d'uso**					
**Snack dolci o salati confezionati**	○	○	○	○	○	○	○	○	○	___	**30g** (es. 1 barretta al cioccolato/snack, 1 sacchetto di patatine piccolo)	○ 0,5	○ 1,0	○ 1,5	○ 2,0	○ 2,5	○ 3,0
**Yogurt vegetali** (es. yogurt a base di soia)	○	○	○	○	○	○	○	○	○	___	**125g (**1 bicchiere piccolo, ½ tazza)	○ 0,5	○ 1,0	○ 1,5	○ 2,0	○ 2,5	○ 3,0
**Sostituti vegetali dei formaggi** (es. tofu)	○	○	○	○	○	○	○	○	○	___	**100g** (1 porzione)	○ 0,5	○ 1,0	○ 1,5	○ 2,0	○ 2,5	○ 3,0
**Sostituti vegetali della carne** (es. burger vegetali)	○	○	○	○	○	○	○	○	○	___	**100g** (es. 1 burger, 1 cotoletta)	○ 0,5	○ 1,0	○ 1,5	○ 2,0	○ 2,5	○ 3,0
**Bevande, frullati e altri prodotti sostitutivi del pasto (incluse barrette) anche in polvere**	○	○	○	○	○	○	○	○	○	___	**125ml** (1 bicchiere piccolo)	○ 0,5	○ 1,0	○ 1,5	○ 2,0	○ 2,5	○ 3,0

Se ci sono altri alimenti che consuma abitualmente ma che non ha trovato nella lista, li inserisca nello spazio sottostante indicandone anche le quantità consumate

___	○	○	○	○	○	○	○	○	○	___	___	○ 0,5	○ 1,0	○ 1,5	○ 2,0	○ 2,5	○ 3,0
___	○	○	○	○	○	○	○	○	○	___	___	○ 0,5	○ 1,0	○ 1,5	○ 2,0	○ 2,5	○ 3,0

___	○	○	○	○	○	○	○	○	○	___	___	○ 0,5	○ 1,0	○ 1,5	○ 2,0	○ 2,5	○ 3,0
___	○	○	○	○	○	○	○	○	○	___	___	○ 0,5	○ 1,0	○ 1,5	○ 2,0	○ 2,5	○ 3,0
___	○	○	○	○	○	○	○	○	○	___	___	○ 0,5	○ 1,0	○ 1,5	○ 2,0	○ 2,5	○ 3,0
___	○	○	○	○	○	○	○	○	○	___	___	○ 0,5	○ 1,0	○ 1,5	○ 2,0	○ 2,5	○ 3,0

Grazie per il tuo prezioso contributo!

Ci piacerebbe chiederti un piccolo ulteriore sforzo: ti invitiamo a completare una breve sezione dedicata alle informazioni sociodemografiche. Questi dati ci aiuteranno a comprendere meglio il contesto delle risposte e ad arricchire l’analisi complessiva del questionario.

La compilazione richiederà solo pochi secondi! Ti ringraziamo ancora per il tuo tempo e la tua disponibilità.

Informazioni sociodemografiche:

Indica il tuo peso (kg)______________________ **Indica la tua altezza (cm)**________________________

Qual è il tuo titolo di studio?
Indica la tua risposta barrando chiaramente una sola opzione tra quelle disponibili

☐ Licenza elementare
☐ Licenza media inferiore
☐ Diploma
☐ Laurea
☐ Post laurea
☐ Non so / Non voglio rispondere

Qual è il reddito familiare annuo lordo?
Indica la tua risposta barrando chiaramente una sola opzione tra quelle disponibili

☐ Meno di 10.000 €
☐ Tra 10.000 € e 20.000 €
☐ Tra 20.000 € e 30.000 €
☐ Tra 30.000 € e 50.000 €
☐ Oltre 50.000 €
☐ Non so / Non voglio rispondere

Indica il numero totale di individui (te compreso) che abitano nella casa in cui vivi attualmente? ________________

Hai finito!!!!

Ti ringraziamo sinceramente per aver dedicato il tuo tempo a completare questo questionario. Il tuo contributo è davvero importante per noi.
