# Supplementary material for: Reproducibility and Validity of a Nova-Based Food Frequency Questionnaire in Older Italian Adults: The NFFQ-Elderly
Source: Nutrients. 2026 Apr 16;18(8):1266. doi: 10.3390/nu18081266 (PMC13118279; doi:10.3390/nu18081266)
Supplement: Supplementary file 1 [file nutrients-18-01266-s001.zip › Table S2 b NFFQ-Elderly.pdf]

Dear participant,
We request a few minutes of your time to complete this questionnaire, which will assist us in better understanding the consumption of ultra-processed foods within the Italian population.
The completion of this questionnaire is part of the NUTRAGE project, "NUTRITION, FEEDING & ACTIVE AGING". NUTRAGE is a multidisciplinary project, promoted and developed by the National Research Council and financed by FOE 2021 funds, aiming to identify optimal dietary regimens capable of preventing or delaying the onset of major neurodegenerative and metabolic pathologies associated with aging, and to formulate specific personalized nutrition programs for the elderly.

When completing the questionnaire, we ask you to evaluate your dietary habits over the course of the previous year.

For each food item, assess both the frequency of consumption and the average quantities consumed per eating occasion.

Below is an example detailing how to complete the questionnaire:
✓ If you consume a food item "Never or less than once a month", it is not necessary to indicate the quantity;
✓ If you consume a food item more frequently than once a month, please also mark the box corresponding to the quantity consumed in terms of average portion size per eating occasion;
✓ If you consume a food item on a daily basis, specify numerically the number of times per day it was consumed.

If you utilize food items to prepare specific recipes (which are not listed), consider the individual ingredients within their respective sections and indicate the frequency and quantities consumed. Should you not find certain foods that you routinely consume or utilize in the list, there is a section on the final page where you may insert them.

If you have altered your diet for health or personal reasons for brief periods, please remember to complete the questionnaire in reference to your habitual dietary intake.

THANK YOU FOR YOUR COLLABORATION!!

Category	Food Item	Consumption Frequency (Select one)	Daily Frequency (If daily)	Reference Serving Size	Serving Size per Occasion (Select one)
FRUIT AND NUTS	Fruit (fresh, cut, blended, frozen)	Never or < 1/month; 1-3/month; 1/week; 2/week; 3/week; 4/week; 5/week; 6/week; Every day	___ times/day	150g (e.g., 1 medium fruit: apple, pear, orange; 2 small fruits: apricots, mandarins, etc.)	0.5; 1.0; 1.5; 2.0; 2.5; 3.0
	100% Fruit juices (fresh or pasteurized, no added sugar/ingredients)	Never or < 1/month; 1-3/month; 1/week; 2/week; 3/week; 4/week; 5/week; 6/week; Every day	___ times/day	200ml (1 medium water glass)	0.5; 1.0; 1.5; 2.0; 2.5; 3.0
	Fruit juices/nectars (with added sugar or other ingredients)	Never or < 1/month; 1-3/month; 1/week; 2/week; 3/week; 4/week; 5/week; 6/week; Every day	___ times/day	200ml (1 medium water glass)	0.5; 1.0; 1.5; 2.0; 2.5; 3.0
	Fruit in syrup	Never or < 1/month; 1-3/month; 1/week; 2/week; 3/week; 4/week; 5/week; 6/week; Every day	___ times/day	150g (e.g., 3 peach halves in syrup)	0.5; 1.0; 1.5; 2.0; 2.5; 3.0
	Dried fruit (e.g., apricots, prunes, figs, dates)	Never or < 1/month; 1-3/month; 1/week; 2/week; 3/week; 4/week; 5/week; 6/week; Every day	___ times/day	30g (e.g., 3 dried figs/dates, 2 level tbsp raisins)	0.5; 1.0; 1.5; 2.0; 2.5; 3.0

	Nuts and seeds (no added salt, sugar, or other ingredients)	Never or < 1/month; 1-3/month; 1/week; 2/week; 3/week; 4/week; 5/week; 6/week; Every day	___ times/day	30g (e.g., 7-8 walnuts, 15-20 almonds/hazelnuts, 3 tbsp sunflower seeds)	0.5; 1.0; 1.5; 2.0; 2.5; 3.0
	Nuts and seeds (with added salt, sugar, or other ingredients)	Never or < 1/month; 1-3/month; 1/week; 2/week; 3/week; 4/week; 5/week; 6/week; Every day	___ times/day	30g (e.g., 7-8 walnuts, 15-20 almonds/hazelnuts, 3 level tbsp sunflower seeds)	0.5; 1.0; 1.5; 2.0; 2.5; 3.0
	Homemade fruit purée (homogenized)	Never or < 1/month; 1-3/month; 1/week; 2/week; 3/week; 4/week; 5/week; 6/week; Every day	___ times/day	80g (e.g., 1 jar)	0.5; 1.0; 1.5; 2.0; 2.5; 3.0
	Packaged fruit purée (homogenized)	Never or < 1/month; 1-3/month; 1/week; 2/week; 3/week; 4/week; 5/week; 6/week; Every day	___ times/day	80g (e.g., 1 jar)	0.5; 1.0; 1.5; 2.0; 2.5; 3.0
	Table olives	Never or < 1/month; 1-3/month; 1/week; 2/week; 3/week; 4/week; 5/week; 6/week; Every day	___ times/day	35g (e.g., 5 olives)	0.5; 1.0; 1.5; 2.0; 2.5; 3.0
VEGETABLES AND LEGUMES	Fresh or frozen plain vegetables (raw or cooked)	Never or < 1/month; 1-3/month; 1/week; 2/week; 3/week; 4/week; 5/week; 6/week; Every day	___ times/day	200g (2-3 tomatoes, 1 fennel, ½ plate spinach / 80g salad e.g. 1 large 500ml bowl)	0.5; 1.0; 1.5; 2.0; 2.5; 3.0
	Ready-to-use/cook packaged vegetables	Never or < 1/month; 1-3/month; 1/week; 2/week; 3/week; 4/week; 5/week; 6/week; Every day	___ times/day	200g (½ plate spinach with cheese)	0.5; 1.0; 1.5; 2.0; 2.5; 3.0

	100% Vegetable juices (fresh or pasteurized, no added sugar/ingredients)	Never or < 1/month; 1-3/month; 1/week; 2/week; 3/week; 4/week; 5/week; 6/week; Every day	___ times/day	200ml (1 medium glass)	0.5; 1.0; 1.5; 2.0; 2.5; 3.0
	Canned or bottled vegetables (e.g., tomato sauce, pickles, in oil)	Never or < 1/month; 1-3/month; 1/week; 2/week; 3/week; 4/week; 5/week; 6/week; Every day	___ times/day	150g (e.g., 1 small glass tomato sauce, 10 med. pickles) / 50g (e.g., 2-3 artichokes in oil)	0.5; 1.0; 1.5; 2.0; 2.5; 3.0
	Fresh or dried legumes (e.g., beans, peas, lentils, etc.)	Never or < 1/month; 1-3/month; 1/week; 2/week; 3/week; 4/week; 5/week; 6/week; Every day	___ times/day	150g (½ plate fresh) / 50g (3-4 tbsp dried)	0.5; 1.0; 1.5; 2.0; 2.5; 3.0
	Canned legumes (with added salt or other ingredients)	Never or < 1/month; 1-3/month; 1/week; 2/week; 3/week; 4/week; 5/week; 6/week; Every day	___ times/day	150g (e.g., 1 small tin)	0.5; 1.0; 1.5; 2.0; 2.5; 3.0
	Ready-to-use canned legumes (e.g., cacciatora, Mexican style, frijoles)	Never or < 1/month; 1-3/month; 1/week; 2/week; 3/week; 4/week; 5/week; 6/week; Every day	___ times/day	200g (e.g., 1 small tin)	0.5; 1.0; 1.5; 2.0; 2.5; 3.0
	Homemade vegetable purée (homogenized)	Never or < 1/month; 1-3/month; 1/week; 2/week; 3/week; 4/week; 5/week; 6/week; Every day	___ times/day	80g (e.g., 1 jar)	0.5; 1.0; 1.5; 2.0; 2.5; 3.0
	Packaged vegetable purée (homogenized)	Never or < 1/month; 1-3/month; 1/week; 2/week; 3/week; 4/week; 5/week; 6/week; Every day	___ times/day	80g (e.g., 1 jar)	0.5; 1.0; 1.5; 2.0; 2.5; 3.0

CEREALS AND TUBERS	Whole grains (e.g., rice, spelt, barley, oats, wheat, etc.)	Never or < 1/month; 1-3/month; 1/week; 2/week; 3/week; 4/week; 5/week; 6/week; Every day	___ times/day	80g (1 medium plate)	0.5; 1.0; 1.5; 2.0; 2.5; 3.0
	Dry/fresh pasta, polenta, couscous, semolina	Never or < 1/month; 1-3/month; 1/week; 2/week; 3/week; 4/week; 5/week; 6/week; Every day	___ times/day	80g dry / 125g fresh (1 medium plate)	0.5; 1.0; 1.5; 2.0; 2.5; 3.0
	Homemade/artisan stuffed pasta (e.g., ravioli, lasagna, potato gnocchi)	Never or < 1/month; 1-3/month; 1/week; 2/week; 3/week; 4/week; 5/week; 6/week; Every day	___ times/day	200g (approx. 1 medium plate)	0.5; 1.0; 1.5; 2.0; 2.5; 3.0
	Packaged ready-to-use stuffed pasta (e.g., ravioli, lasagna, gnocchi, risotto)	Never or < 1/month; 1-3/month; 1/week; 2/week; 3/week; 4/week; 5/week; 6/week; Every day	___ times/day	200g (approx. 1 medium plate)	0.5; 1.0; 1.5; 2.0; 2.5; 3.0
	Instant noodles, rice, or soups	Never or < 1/month; 1-3/month; 1/week; 2/week; 3/week; 4/week; 5/week; 6/week; Every day	___ times/day	90g (1 serving)	0.5; 1.0; 1.5; 2.0; 2.5; 3.0
	Homemade/artisan fresh bread and rolls	Never or < 1/month; 1-3/month; 1/week; 2/week; 3/week; 4/week; 5/week; 6/week; Every day	___ times/day	50g (e.g., 1 medium slice, 1 small roll)	0.5; 1.0; 1.5; 2.0; 2.5; 3.0

	Packaged bread and rolls (e.g., sliced bread)	Never or < 1/month; 1-3/month; 1/week; 2/week; 3/week; 4/week; 5/week; 6/week; Every day	___ times/day	50g (e.g., 2-3 slices, 1 small roll)	0.5; 1.0; 1.5; 2.0; 2.5; 3.0
	Bread substitutes (e.g., crackers, taralli, breadsticks, rusks)	Never or < 1/month; 1-3/month; 1/week; 2/week; 3/week; 4/week; 5/week; 6/week; Every day	___ times/day	30g (e.g., 1 pack crackers, 3-4 tarallini, 1 frisella, 3-4 rusks)	0.5; 1.0; 1.5; 2.0; 2.5; 3.0
	Packaged sandwiches	Never or < 1/month; 1-3/month; 1/week; 2/week; 3/week; 4/week; 5/week; 6/week; Every day	___ times/day	80g (e.g., 1 sandwich)	0.5; 1.0; 1.5; 2.0; 2.5; 3.0
	Homemade/artisan pizza, focaccia	Never or < 1/month; 1-3/month; 1/week; 2/week; 3/week; 4/week; 5/week; 6/week; Every day	___ times/day	350g (1 pizza)	0.5; 1.0; 1.5; 2.0; 2.5; 3.0
	Packaged ready-to-use pizza, focaccia (e.g., frozen)	Never or < 1/month; 1-3/month; 1/week; 2/week; 3/week; 4/week; 5/week; 6/week; Every day	___ times/day	350g (1 pizza)	0.5; 1.0; 1.5; 2.0; 2.5; 3.0
	Homemade/artisan savory pies, pastries	Never or < 1/month; 1-3/month; 1/week; 2/week; 3/week; 4/week; 5/week; 6/week; Every day	___ times/day	150g (1 medium slice)	0.5; 1.0; 1.5; 2.0; 2.5; 3.0
	Packaged savory pies, pastries	Never or < 1/month; 1-3/month; 1/week; 2/week; 3/week; 4/week; 5/week; 6/week; Every day	___ times/day	150g (1 medium slice)	0.5; 1.0; 1.5; 2.0; 2.5; 3.0

	Plain breakfast cereals or muesli with nuts/dried fruit (no added sugar)	Never or < 1/month; 1-3/month; 1/week; 2/week; 3/week; 4/week; 5/week; 6/week; Every day	___ times/day	30g (e.g., 6-8 tbsp cornflakes, 5-6 tbsp heavier flakes, 3 tbsp muesli)	0.5; 1.0; 1.5; 2.0; 2.5; 3.0
	Breakfast cereals, muesli/granola with added sugar/ingredients, cereal bars	Never or < 1/month; 1-3/month; 1/week; 2/week; 3/week; 4/week; 5/week; 6/week; Every day	___ times/day	30g (e.g., 6-8 tbsp cornflakes, 5-6 tbsp heavier flakes, 3 tbsp muesli)	0.5; 1.0; 1.5; 2.0; 2.5; 3.0
	Homemade/artisan potatoes, croquettes, potato gateau	Never or < 1/month; 1-3/month; 1/week; 2/week; 3/week; 4/week; 5/week; 6/week; Every day	___ times/day	200g (2 small potatoes)	0.5; 1.0; 1.5; 2.0; 2.5; 3.0
	Ready-to-use/cook packaged potatoes, croquettes, etc.	Never or < 1/month; 1-3/month; 1/week; 2/week; 3/week; 4/week; 5/week; 6/week; Every day	___ times/day	150g (1 medium plate)	0.5; 1.0; 1.5; 2.0; 2.5; 3.0
MEAT AND FISH	Fresh/frozen meat and poultry (plain steaks, fillets, etc.)	Never or < 1/month; 1-3/month; 1/week; 2/week; 3/week; 4/week; 5/week; 6/week; Every day	___ times/day	100g (e.g., 1 slice meat, 1 small chicken drumstick)	0.5; 1.0; 1.5; 2.0; 2.5; 3.0
	Cold cuts, cured meats, smoked meat	Never or < 1/month; 1-3/month; 1/week; 2/week; 3/week; 4/week; 5/week; 6/week; Every day	___ times/day	50g (e.g., 3-4 slices ham, 5-6 slices salami or bresaola)	0.5; 1.0; 1.5; 2.0; 2.5; 3.0
	Homemade cutlets, sausages, burgers	Never or < 1/month; 1-3/month; 1/week; 2/week; 3/week; 4/week; 5/week; 6/week; Every day	___ times/day	100g (e.g., 1 sausage, 1 burger)	0.5; 1.0; 1.5; 2.0; 2.5; 3.0

	Packaged ready-to-use/cook cutlets, nuggets, meat sticks	Never or < 1/month; 1-3/month; 1/week; 2/week; 3/week; 4/week; 5/week; 6/week; Every day	___ times/day	100g (e.g., 1 cutlet, 5-6 chicken nuggets)	0.5; 1.0; 1.5; 2.0; 2.5; 3.0
	Ready-to-use/cook packaged sausages, hot dogs, burgers, canned meat	Never or < 1/month; 1-3/month; 1/week; 2/week; 3/week; 4/week; 5/week; 6/week; Every day	___ times/day	100g (e.g., 1 sausage, 1 burger, 4 small hot dogs)	0.5; 1.0; 1.5; 2.0; 2.5; 3.0
	Fresh/frozen fish, crustaceans, mollusks	Never or < 1/month; 1-3/month; 1/week; 2/week; 3/week; 4/week; 5/week; 6/week; Every day	___ times/day	150g (e.g., 1 small fish, 1 medium fillet, 3 prawns, 25 mussels)	0.5; 1.0; 1.5; 2.0; 2.5; 3.0
	Smoked, dried, or brined fish	Never or < 1/month; 1-3/month; 1/week; 2/week; 3/week; 4/week; 5/week; 6/week; Every day	___ times/day	50g (e.g., 4-5 thin slices smoked salmon, ½ salt cod fillet)	0.5; 1.0; 1.5; 2.0; 2.5; 3.0
	Canned/jarred fish in water (plain)	Never or < 1/month; 1-3/month; 1/week; 2/week; 3/week; 4/week; 5/week; 6/week; Every day	___ times/day	50g (e.g., 1 small tin of tuna)	0.5; 1.0; 1.5; 2.0; 2.5; 3.0
	Canned/jarred fish in oil	Never or < 1/month; 1-3/month; 1/week; 2/week; 3/week; 4/week; 5/week; 6/week; Every day	___ times/day	50g (e.g., 1 small tin of tuna)	0.5; 1.0; 1.5; 2.0; 2.5; 3.0
	Homemade fish cutlets, fish sticks	Never or < 1/month; 1-3/month; 1/week; 2/week; 3/week; 4/week; 5/week; 6/week; Every day	___ times/day	100g (e.g., 1 cutlet, 4 fish sticks)	0.5; 1.0; 1.5; 2.0; 2.5; 3.0

	Ready-to-use/cook packaged fish cutlets, sticks, nuggets	Never or < 1/month; 1-3/month; 1/week; 2/week; 3/week; 4/week; 5/week; 6/week; Every day	___ times/day	100g (e.g., 4 fish sticks)	0.5; 1.0; 1.5; 2.0; 2.5; 3.0
	Packaged ready meals (meat/fish) (e.g., mussels in tomato, marinated salmon, seafood salad)	Never or < 1/month; 1-3/month; 1/week; 2/week; 3/week; 4/week; 5/week; 6/week; Every day	___ times/day	1 serving	0.5; 1.0; 1.5; 2.0; 2.5; 3.0
MILK, DAIRY, AND EGGS	Cow, sheep, or goat milk (pasteurized or UHT)	Never or < 1/month; 1-3/month; 1/week; 2/week; 3/week; 4/week; 5/week; 6/week; Every day	___ times/day	125ml (e.g., 1 small glass, ½ medium cup)	0.5; 1.0; 1.5; 2.0; 2.5; 3.0
	Plant-based milk (e.g., soy, almond, oat, hazelnut)	Never or < 1/month; 1-3/month; 1/week; 2/week; 3/week; 4/week; 5/week; 6/week; Every day	___ times/day	125ml (e.g., 1 small glass, ½ medium cup)	0.5; 1.0; 1.5; 2.0; 2.5; 3.0
	Plain yogurt (no added sugar)	Never or < 1/month; 1-3/month; 1/week; 2/week; 3/week; 4/week; 5/week; 6/week; Every day	___ times/day	125ml (e.g., 1 pot)	0.5; 1.0; 1.5; 2.0; 2.5; 3.0
	Fruit/flavored yogurt, fermented milk, kefir	Never or < 1/month; 1-3/month; 1/week; 2/week; 3/week; 4/week; 5/week; 6/week; Every day	___ times/day	125ml (e.g., 1 pot, 1 bottle)	0.5; 1.0; 1.5; 2.0; 2.5; 3.0

	Ricotta (cow or sheep)	Never or < 1/month; 1-3/month; 1/week; 2/week; 3/week; 4/week; 5/week; 6/week; Every day	___ times/day	100g (1 serving)	0.5; 1.0; 1.5; 2.0; 2.5; 3.0
	Soft cheeses (including in sandwiches)	Never or < 1/month; 1-3/month; 1/week; 2/week; 3/week; 4/week; 5/week; 6/week; Every day	___ times/day	100g (e.g., 1 small mozzarella)	0.5; 1.0; 1.5; 2.0; 2.5; 3.0
	Hard cheeses (including in sandwiches)	Never or < 1/month; 1-3/month; 1/week; 2/week; 3/week; 4/week; 5/week; 6/week; Every day	___ times/day	50g (e.g., 2 slices pecorino)	0.5; 1.0; 1.5; 2.0; 2.5; 3.0
	Processed/spreadable cheeses (including in sandwiches)	Never or < 1/month; 1-3/month; 1/week; 2/week; 3/week; 4/week; 5/week; 6/week; Every day	___ times/day	30g (1 slice, 1 triangle) / 80g spread	0.5; 1.0; 1.5; 2.0; 2.5; 3.0
	Grated seasoning cheese (e.g., Grana, Parmesan, Pecorino)	Never or < 1/month; 1-3/month; 1/week; 2/week; 3/week; 4/week; 5/week; 6/week; Every day	___ times/day	10g (1 tbsp)	0.5; 1.0; 1.5; 2.0; 2.5; 3.0
	Cream	Never or < 1/month; 1-3/month; 1/week; 2/week; 3/week; 4/week; 5/week; 6/week; Every day	___ times/day	10ml (1 tbsp)	0.5; 1.0; 1.5; 2.0; 2.5; 3.0
	Eggs (including in omelets or sandwiches)	Never or < 1/month; 1-3/month; 1/week; 2/week; 3/week; 4/week; 5/week; 6/week; Every day	___ times/day	50g (1 egg)	0.5; 1.0; 1.5; 2.0; 2.5; 3.0

OILS, FATS, AND CONDIMENTS	Olive oil, extra virgin olive oil	Never or < 1/month; 1-3/month; 1/week; 2/week; 3/week; 4/week; 5/week; 6/week; Every day	___ times/day	10ml (1 tbsp)	0.5; 1.0; 1.5; 2.0; 2.5; 3.0
	Seed oil	Never or < 1/month; 1-3/month; 1/week; 2/week; 3/week; 4/week; 5/week; 6/week; Every day	___ times/day	10ml (1 tbsp)	0.5; 1.0; 1.5; 2.0; 2.5; 3.0
	Butter, lard, tallow	Never or < 1/month; 1-3/month; 1/week; 2/week; 3/week; 4/week; 5/week; 6/week; Every day	___ times/day	10g (½ knob, 1 hotel pack)	0.5; 1.0; 1.5; 2.0; 2.5; 3.0
	Margarine	Never or < 1/month; 1-3/month; 1/week; 2/week; 3/week; 4/week; 5/week; 6/week; Every day	___ times/day	10g (½ knob, 1 hotel pack)	0.5; 1.0; 1.5; 2.0; 2.5; 3.0
	Ready-made sauces (e.g., mayo, ketchup)	Never or < 1/month; 1-3/month; 1/week; 2/week; 3/week; 4/week; 5/week; 6/week; Every day	___ times/day	18g (1 heaping tbsp)	0.5; 1.0; 1.5; 2.0; 2.5; 3.0
	Homemade pasta sauces (e.g., pesto, ragù)	Never or < 1/month; 1-3/month; 1/week; 2/week; 3/week; 4/week; 5/week; 6/week; Every day	___ times/day	50g (1 serving)	0.5; 1.0; 1.5; 2.0; 2.5; 3.0
	Ready-made pasta sauces (e.g., pesto, ragù)	Never or < 1/month; 1-3/month; 1/week; 2/week; 3/week; 4/week; 5/week; 6/week; Every day	___ times/day	50g (1 serving)	0.5; 1.0; 1.5; 2.0; 2.5; 3.0

SWEETS AND SWEETENERS	Homemade/artisan biscuits	Never or < 1/month; 1-3/month; 1/week; 2/week; 3/week; 4/week; 5/week; 6/week; Every day	____ times/day	30g (e.g., 2-3 shortbread, 4-5 dry biscuits)	0.5; 1.0; 1.5; 2.0; 2.5; 3.0
	Packaged biscuits	Never or < 1/month; 1-3/month; 1/week; 2/week; 3/week; 4/week; 5/week; 6/week; Every day	____ times/day	30g (e.g., 2-3 shortbread, 4-5 dry biscuits)	0.5; 1.0; 1.5; 2.0; 2.5; 3.0
	Homemade/artisan cakes, desserts	Never or < 1/month; 1-3/month; 1/week; 2/week; 3/week; 4/week; 5/week; 6/week; Every day	____ times/day	100g (1 small slice, 1 bowl)	0.5; 1.0; 1.5; 2.0; 2.5; 3.0
	Packaged cakes, desserts, or dessert mixes	Never or < 1/month; 1-3/month; 1/week; 2/week; 3/week; 4/week; 5/week; 6/week; Every day	____ times/day	100g (1 small slice, 1 bowl)	0.5; 1.0; 1.5; 2.0; 2.5; 3.0
	Packaged croissants, snacks	Never or < 1/month; 1-3/month; 1/week; 2/week; 3/week; 4/week; 5/week; 6/week; Every day	____ times/day	50g (1 piece)	0.5; 1.0; 1.5; 2.0; 2.5; 3.0
	Fresh/artisan gelato	Never or < 1/month; 1-3/month; 1/week; 2/week; 3/week; 4/week; 5/week; 6/week; Every day	____ times/day	100g (1 serving)	0.5; 1.0; 1.5; 2.0; 2.5; 3.0
	Packaged ice cream (cone, stick, tub)	Never or < 1/month; 1-3/month; 1/week; 2/week; 3/week; 4/week; 5/week; 6/week; Every day	____ times/day	70g (1 cone, 1 stick, 1 bowl)	0.5; 1.0; 1.5; 2.0; 2.5; 3.0

	Chocolate	Never or < 1/month; 1-3/month; 1/week; 2/week; 3/week; 4/week; 5/week; 6/week; Every day	___ times/day	30g (e.g., 2-3 chocolates)	0.5; 1.0; 1.5; 2.0; 2.5; 3.0
	Candies	Never or < 1/month; 1-3/month; 1/week; 2/week; 3/week; 4/week; 5/week; 6/week; Every day	___ times/day	10g (e.g., 2-3 candies)	0.5; 1.0; 1.5; 2.0; 2.5; 3.0
	Spreadable creams (e.g., hazelnut, pistachio)	Never or < 1/month; 1-3/month; 1/week; 2/week; 3/week; 4/week; 5/week; 6/week; Every day	___ times/day	15g (1 tbsp)	0.5; 1.0; 1.5; 2.0; 2.5; 3.0
	Homemade jams/preserves	Never or < 1/month; 1-3/month; 1/week; 2/week; 3/week; 4/week; 5/week; 6/week; Every day	___ times/day	20g (2 tbsp)	0.5; 1.0; 1.5; 2.0; 2.5; 3.0
	Packaged jams/preserves	Never or < 1/month; 1-3/month; 1/week; 2/week; 3/week; 4/week; 5/week; 6/week; Every day	___ times/day	20g (2 tbsp)	0.5; 1.0; 1.5; 2.0; 2.5; 3.0
	Sugar, fructose, molasses	Never or < 1/month; 1-3/month; 1/week; 2/week; 3/week; 4/week; 5/week; 6/week; Every day	___ times/day	5g (1 tsp)	0.5; 1.0; 1.5; 2.0; 2.5; 3.0
	Honey, maple syrup	Never or < 1/month; 1-3/month; 1/week; 2/week; 3/week; 4/week; 5/week; 6/week; Every day	___ times/day	10g (1 tsp)	0.5; 1.0; 1.5; 2.0; 2.5; 3.0

	Sweeteners (e.g., stevia, aspartame, saccharin)	Never or < 1/month; 1-3/month; 1/week; 2/week; 3/week; 4/week; 5/week; 6/week; Every day	___ times/day	1 sachet, 1 tablet, 2 drops	0.5; 1.0; 1.5; 2.0; 2.5; 3.0
BEVERAGES	Tea, infusions, herbal tea	Never or < 1/month; 1-3/month; 1/week; 2/week; 3/week; 4/week; 5/week; 6/week; Every day	___ times/day	125ml (1 small cup)	0.5; 1.0; 1.5; 2.0; 2.5; 3.0
	Coffee	Never or < 1/month; 1-3/month; 1/week; 2/week; 3/week; 4/week; 5/week; 6/week; Every day	___ times/day	30ml (1 small espresso cup)	0.5; 1.0; 1.5; 2.0; 2.5; 3.0
	Soft drinks (e.g., iced tea, orange soda, cola, sports drinks, non-alcoholic aperitifs)	Never or < 1/month; 1-3/month; 1/week; 2/week; 3/week; 4/week; 5/week; 6/week; Every day	___ times/day	330ml (1 can)	0.5; 1.0; 1.5; 2.0; 2.5; 3.0
	Energy drinks	Never or < 1/month; 1-3/month; 1/week; 2/week; 3/week; 4/week; 5/week; 6/week; Every day	___ times/day	250ml (1 can)	0.5; 1.0; 1.5; 2.0; 2.5; 3.0
	Cocoa-based drinks (e.g., hot chocolate)	Never or < 1/month; 1-3/month; 1/week; 2/week; 3/week; 4/week; 5/week; 6/week; Every day	___ times/day	125ml (1 small glass)	0.5; 1.0; 1.5; 2.0; 2.5; 3.0
	Wine	Never or < 1/month; 1-3/month; 1/week; 2/week; 3/week; 4/week; 5/week; 6/week; Every day	___ times/day	125ml (1 small glass)	0.5; 1.0; 1.5; 2.0; 2.5; 3.0

	Beer	Never or < 1/month; 1-3/month; 1/week; 2/week; 3/week; 4/week; 5/week; 6/week; Every day	___ times/day	330ml (1 can)	0.5; 1.0; 1.5; 2.0; 2.5; 3.0
	Other spirits (e.g., bitters, rum, gin, grappa)	Never or < 1/month; 1-3/month; 1/week; 2/week; 3/week; 4/week; 5/week; 6/week; Every day	___ times/day	40ml	0.5; 1.0; 1.5; 2.0; 2.5; 3.0
OTHER	Packaged sweet or savory snacks	Never or < 1/month; 1-3/month; 1/week; 2/week; 3/week; 4/week; 5/week; 6/week; Every day	___ times/day	30g (e.g., 1 cereal bar/snack, 1 small bag of chips)	0.5; 1.0; 1.5; 2.0; 2.5; 3.0
	Plant-based yogurt (e.g., soy yogurt)	Never or < 1/month; 1-3/month; 1/week; 2/week; 3/week; 4/week; 5/week; 6/week; Every day	___ times/day	125g (1 small glass, ½ cup)	0.5; 1.0; 1.5; 2.0; 2.5; 3.0
	Plant-based cheese substitutes (e.g., tofu)	Never or < 1/month; 1-3/month; 1/week; 2/week; 3/week; 4/week; 5/week; 6/week; Every day	___ times/day	100g (1 serving)	0.5; 1.0; 1.5; 2.0; 2.5; 3.0
	Plant-based meat substitutes (e.g., veggie burgers)	Never or < 1/month; 1-3/month; 1/week; 2/week; 3/week; 4/week; 5/week; 6/week; Every day	___ times/day	100g (e.g., 1 burger, 1 cutlet)	0.5; 1.0; 1.5; 2.0; 2.5; 3.0
	Meal replacement beverages/shakes/bars (incl. powder)	Never or < 1/month; 1-3/month; 1/week; 2/week; 3/week; 4/week; 5/week; 6/week; Every day	___ times/day	125ml (1 small glass)	0.5; 1.0; 1.5; 2.0; 2.5; 3.0

SOCIODEMOGRAPHIC INFORMATION

- Weight (kg): _______
- Height (cm): _______
- Educational Attainment:
  - Primary school certificate
  - Lower secondary school certificate
  - High school diploma
  - Bachelor's Degree
  - Postgraduate degree
- Gross Annual Household Income:
  - Less than 10,000 €
  - Between 10,000 € and 20,000 €
  - Between 20,000 € and 30,000 €
  - Between 30,000 € and 50.000 €
  - Over 50,000 €
- Number of Household Members: _______

Methodological Note: The present English translation of the NFFQ-Elderly is provided for international accessibility. The current validation applies exclusively to the Italian demographic. Future studies aiming to utilize this tool in different cultural or linguistic contexts must perform proper cross-cultural adaptation and validation procedures.
