# Supplementary material for: Reproducibility and Validity of a Nova-Based Food Frequency Questionnaire in Older Italian Adults: The NFFQ-Elderly
Source: Nutrients. 2026 Apr 16;18(8):1266. doi: 10.3390/nu18081266 (PMC13118279; doi:10.3390/nu18081266)
Supplement: Supplementary file 1 [file nutrients-18-01266-s001.zip › Tables S3 and S4 (Supplementary).pdf]

Table S3. Questionnaire validity in women (n = 63).

	Three-day food records	NFFQ T0	r	*p*-value	ICC	Lower limit 95% CI	Upper limit 95% CI
Nova groups (g/d)							
MPFs	676.5 (202.2)	831.7 (221.5)	0.50	<.0001	0.33	0.15	0.57
PCIs	37.2 (12.1)	31.5 (11.4)	0.19	0.14	0.00	0.00	1.00
PFs	163.5 (86.5)	295.6 (145.7)	0.34	0.0065	-	-	-
PCIs + PFs	200.7 (87.7)	327.1 (145.4)	0.39	0.0016	-	-	-
UPFs	111.4 (90.1)	215.7 (188.2)	0.41	0.0007	0.16	0.03	0.53
Total food intake	988.6 (211.9)	1374.5 (324.5)	0.40	0.010	-	-	-
Nova groups (energy ratio)							
MPFs	37.7 (10.2)	31.4 (8.8)	0.47	0.0001	0.29	0.12	0.55
PCIs	20.6 (5.5)	14.0 (4.9)	0.081	0.53	-	-	-
PFs	23.8 (9.9)	14.0 (4.9)	0.18	0.16	0.06	0.00	0.85
PCIs + PFs	44.4 (9.6)	44.4 (10.0)	0.30	0.018	0.23	0.07	0.53
UPFs	17.9 (11.5)	24.2 (12.4)	0.37	0.0027	0.32	0.14	0.57
Total energy intake (kcal/d)	1479.5 (294.5)	1890.9 (519.2)	0.33	0.0083	-	-	-
Nova groups (weight ratio)							
MPFs	67.9 (11.3)	61.3 (12.2)	0.51	<.0001	0.34	0.16	0.58
PCIs	3.9 (1.3)	2.4 (0.9)	0.17	0.17	-	-	-
PFs	16.8 (8.4)	21.5 (9.0)	0.39	0.0016	0.29	0.12	0.55
PCIs + PFs	20.7 (8.5)	23.9 (8.8)	0.41	0.0008	0.23	0.07	0.53
UPFs	11.4 (9.3)	14.8 (10.2)	0.40	0.0010	0.45	0.27	0.65

Data are reported as mean ± SD. Abbreviations: MPFs (unprocessed or minimally processed foods); PCIs (processed culinary ingredients); PFs (processed foods); UPFs (ultra-processed foods); CI (confidence interval); ICC (intraclass correlation coefficients); r (Pearson correlation coefficients).

Table S4. Questionnaire validity in men (n = 48).

	Three-day food records	NFFQ T0	r	*p*-value	ICC	Lower limit 95% CI	Upper limit 95% CI
Nova groups (g/d)							
MPFs	717.7 (256.9)	833.6 (281.5)	0.66	<.0001	0.69	0.52	0.82
PCIs	35.5 (13.1)	24.5 (11.1)	0.24	0.099	0.04	0.00	0.99
PFs	281.2 (175.3)	397.3 (197.3)	0.44	0.0018	0.47	0.27	0.68
PCIs + PFs	316.8 (177.5)	421.8 (202.9)	0.44	0.0019	0.51	0.31	0.71
UPFs	99.4 (66.7)	167.9 (108.4)	0.59	<.0001	0.37	0.17	0.62
Total food intake	1133.8 (313.5)	1423.3 (394.7)	0.60	<.0001	0.42	0.21	0.65
Nova groups (energy ratio)							
MPFs	36.6 (10.1)	32.5 (8.3)	0.49	0.0004	0.41	0.21	0.65
PCIs	17.8 (6.1)	10.5 (4.4)	-0.021	0.88	-	-	-
PFs	31.3 (13.6)	36.1 (9.8)	0.37	0.0092	0.28	0.10	0.59
PCIs + PFs	49.1 (12.2)	46.6 (10.4)	0.41	0.0037	0.47	0.27	0.68
UPFs	14.3 (8.3)	20.8 (10.5)	0.56	<.0001	0.42	0.21	0.65
Total energy intake (kcal/d)	1706.2 (415.5)	1917.0 (508.4)	0.54	<.0001	0.47	0.29	0.68
Nova groups (weight ratio)							
MPFs	62.9 (13.2)	58.6 (10.4)	0.71	<.0001	0.65	0.47	0.79
PCIs	3.3 (1.3)	1.8 (0.8)	0.10	0.50	-	-	-
PFs	24.8 (13.6)	29.5 (10.4)	0.56	<.0001	0.61	0.42	0.77
PCIs + PFs	28.1 (13.7)	29.5 (10.4)	0.58	<.0001	0.47	0.27	0.68
UPFs	9.0 (6.0)	11.9 (6.1)	0.54	<.0001	0.48	0.28	0.69

Data are reported as mean ± SD. Abbreviations: MPFs (unprocessed or minimally processed foods); PCIs (processed culinary ingredients); PFs (processed foods); UPFs (ultra-processed foods); CI (confidence interval); ICC (intraclass correlation coefficients); r (Pearson correlation coefficients).
